# Estrogen‐Induced LncRNA, LINC02568, Promotes Estrogen Receptor‐Positive Breast Cancer Development and Drug Resistance Through Both *In* *Trans* and *In Cis* Mechanisms

**DOI:** 10.1002/advs.202206663

**Published:** 2023-07-05

**Authors:** Xue Chen, Jian‐cheng Ding, Guo‐sheng Hu, Xing‐yi Shu, Yan Liu, Jun Du, Zi‐jing Wen, Jun‐yi Liu, Hai‐hua Huang, Guo‐hui Tang, Wen Liu

**Affiliations:** ^1^ State Key Laboratory of Cellular Stress Biology School of Pharmaceutical Sciences Xiamen University Xiang'an South Road Xiamen 361102 Fujian China; ^2^ Fujian Provincial Key Laboratory of Innovative Drug Target Research School of Pharmaceutical Sciences Xiamen University Xiang'an South Road Xiamen 361102 Fujian China; ^3^ Xiang An Biomedicine Laboratory School of Pharmaceutical Sciences Xiamen University Xiang'an South Road Xiamen 361102 Fujian China; ^4^ Department of Anus and Bowels Affiliated Nanhua Hospital University of South China Dongfeng South Road Hengyang 421002 Hunan China; ^5^ State Key Laboratory of Molecular Vaccinology and Molecular Diagnostics National Institute of Diagnostics and Vaccine Development in Infectious Diseases Xiamen University Xiang'an South Road Xiamen 361102 Fujian China; ^6^ Department of Pathology The Second Affiliated Hospital Shantou University Medical College Dongxia North Road Shantou 515041 Guangdong China

**Keywords:** ER‐positive breast cancer, estrogen and estrogen receptor, lncRNA

## Abstract

Endocrine therapy is the frontline treatment for estrogen receptor (ER) positive breast cancer patients. However, the primary and acquired resistance to endocrine therapy drugs remain as a major challenge in the clinic. Here, this work identifies an estrogen‐induced lncRNA, LINC02568, which is highly expressed in ER‐positive breast cancer and functional important in cell growth in vitro and tumorigenesis in vivo as well as endocrine therapy drug resistance. Mechanically, this work demonstrates that LINC02568 regulates estrogen/ER*α*‐induced gene transcriptional activation *in trans* by stabilizing *ESR1* mRNA through sponging miR‐1233‐5p in the cytoplasm. Meanwhile, LINC02568 contributes to tumor‐specific pH homeostasis by regulating carbonic anhydrase CA12 *in cis* in the nucleus. The dual functions of LINC02568 together contribute to breast cancer cell growth and tumorigenesis as well as endocrine therapy drug resistance. Antisense oligonucleotides (ASO) targeting LINC02568 significantly inhibits ER‐positive breast cancer cell growth in vitro and tumorigenesis in vivo. Furthermore, combination treatment with ASO targeting LINC02568 and endocrine therapy drugs or CA12 inhibitor U‐104 exhibits synergistic effects on tumor growth. Taken together, the findings reveal the dual mechanisms of LINC02568 in regulating ER*α* signaling and pH homeostasis in ER‐positive breast cancer, and indicated that targeting LINC02568 might represent a potential therapeutic avenue in the clinic.

## Graphical abstract

Estrogen/ER*α*‐induced LINC02568 acts as a ceRNA to sponge miR‐1233‐5p to regulate the expression of *ESR1* in the cytosol, in turn promoting the activation of ER*α*‐target gene program. LINC02568 also regulates its adjacent coding gene *CA12 in cis* to regulate cellular pH. LINC02568‐regulated ER*α*‐target gene activation and cellular pH both contribute to the development and drug resistance of ER^+^ breast cancer.

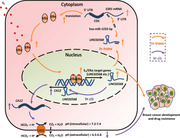

## 1 Introduction

Breast cancer ranks the first in both morbidity and mortality among all cancer types in women worldwide, posing a serious threat to women's health.^[^ ^1^ ^]^ Breast cancer is classified into four different clinically relevant molecular subtypes: luminal A, luminal B, HER2‐enriched, and basal‐like.^[^ ^2^, ^3^, ^4^, ^5^ ^]^ ER‐positive (ER^+^) breast cancer including luminal A and B accounts for over 70% of all types of breast cancer.^[^ ^6^ ^]^ Although a fraction of breast cancers is caused by heritable mutations in oncogenes or tumor suppressor genes, a substantial portion of breast cancers arise from reprogramming of cancer cell genomes due to altered epigenetic mechanisms.^[^ ^7^ ^]^ Therefore, it is of great significance to understand the occurrence and development of ER^+^ breast cancer by dissecting the epigenetic mechanisms involved in, so as to find key regulators.

The primary mechanism in ER^+^ breast cancer is the aberrant activation of ER signaling pathway due to estrogen (17‐*β*‐estradiol, estradiol, E_2_) overexposure. Estrogen effects on breast cancer cells are mediated by ERs including ER*α* and ER*β*, of which ER*α* is thought to be the main driver of ER^+^ breast cancer.^[^ ^8^ ^]^ Endocrine therapy by blocking the function of ER*α* has become the frontline treatment option for patients with ER^+^ breast cancer.^[^ ^5^, ^9^, ^10^ ^]^ This strategy aims to inhibit estrogen‐dependent cell growth by disturbing the biosynthesis of estrogen or reducing ER*α* activity. The endocrine therapy for breast cancer has made great progress with the development of selective estrogen receptor modulators (SERMs), aromatase inhibitors (AIs), selective estrogen receptor degraders (SERDs), and estrogen receptor PROTAC degrader.^[^ ^10^, ^11^, ^12^ ^]^ Though the 5‐year survival rate of ER^+^ breast cancer is impressive, however, there are still around 30% of patients undergo endocrine therapy drug resistance, recurrence, and metastasis. The ligand‐independent reactivation of ER is the common driver of endocrine therapy resistance.^[^ ^13^ ^]^ Therefore, the inhibition of ER itself or ER signaling pathway represent one of the most effective means to overcome resistance.^[^ ^13^ ^]^ As a result, exploring novel strategies targeting key regulators in estrogen signaling pathway may provide effective ways to overcome endocrine therapy drug resistance and new sights for ER^+^ breast cancer therapy.

Epigenetic changes in tumors are orchestrated by a plethora of molecules including nuclear receptors, transcription factors, epigenetic regulators, and noncoding RNAs, among others. LncRNAs are kinds of RNA molecules that are longer than 200 nucleotides (nt) in length and usually without coding potential. As noncoding RNA molecules, the apparent regulatory mechanisms involved in lncRNAs are closely related to their cellular localization. Most of nuclear lncRNAs are enriched in spatial proximity to their transcriptional loci and recruit diffusible RNAs and proteins to drive the formation of nuclear compartments, thereby shaping DNA contacts, heterochromatin, and gene expression *in* *cis*.^[^ ^14^ ^]^ Some lncRNAs in the nucleus leave away from the transcription sites and are involved in nuclear architecture organization, alternative splicing, and gene regulation *in* *trans*.^[^ ^15^, ^16^, ^17^ ^]^ LncRNAs exported to the cytosol can often regulate the protein modification and intercellular signal transduction by forming RNP complexes, or regulating mRNA translation or degradation directly or indirectly by interacting with mRNAs or miRNAs.^[^ ^18^, ^19^, ^20^, ^21^ ^]^ By exercising diverse regulatory roles, lncRNAs are known to be involved in tumor occurrence and development.^[^ ^22^ ^]^ LncRNAs have shown great potential as drug targets. Using of small interfering RNAs (siRNAs) and antisense oligos (ASOs) to recruit RNA‐induced silencing complex (RISC) and RNase H (RNase H), respectively, to target and silence specific RNA molecules presents great promise for RNA therapy.^[^ ^23^, ^24^ ^]^ The development of ribonuclease‐targeting chimeras (RIBOTAC) technology makes the approach of targeting RNAs by small molecules and recruiting ribozymes for degradation another prospective strategy.^[^ ^25^, ^26^, ^27^, ^28^ ^]^ Therefore, developing drugs targeting lncRNAs will provide new avenue for the treatment of ER^+^ breast cancer in clinic.

The change of pH homeostasis inside (pHi) and outside (pHe) cells is one of the important epigenetic‐related changes in the process of tumor development. Tumor cells exhibit increased glucose consumption, altered metabolism, and increased glycolysis ratio, which cause increased lactate production and excretion from cells, resulting in a decrease in pHe and a slightly increase in pHi.^[^ ^29^, ^30^, ^31^ ^]^ The reversed pH gradient in tumor cells provides adaptive advantages for tumor growth, drug resistance, metastasis, and recurrence.^[^ ^29^, ^31^, ^32^, ^33^ ^]^ The altered expression of some key molecules is proved to be responsible for the unique pH gradient in tumor cells, including Na^+^/H^+^ exchanger 1 (NHE1), Vacuolar H^+^ ATPases (V‐ATPases), and carbonic anhydrases (CAs).^[^ ^29^ ^]^ Studies have shown that transmembrane CAs cooperate with cytosolic CAs to create the pH differences in the hypoxic tumor microenvironment, thereby promoting ATP synthesis, cell growth, and other malignant behaviors of tumor cells under acidic conditions.^[^ ^30^, ^34^, ^35^, ^36^, ^37^ ^]^ CA12 belongs to membrane‐associated CAs, and can reversibly catalyze CO_2_ to HCO_3_^−^ and a proton in the presence of water.^[^ ^38^ ^]^ CA12 has been shown to participate in cell signal transduction, survival, migration, invasion, and stemness of tumor cells.^[^ ^39^, ^40^, ^41^ ^]^ Moreover, increased expression of CA12 was observed in drug‐resistant cells.^[^ ^33^ ^]^ However, the underlying regulatory mechanisms of the differential expression of CA12 in tumor cells is still unclear. Understanding of such mechanisms will help us to develop therapeutic strategies to inhibit the formation of tumor‐unique pH environment and therefore tumor growth, drug resistance, metastasis, and recurrence.

In this study, we systematically identified lncRNAs that are induced by estrogen in ER^+^ breast cancer cells through transcriptomic analysis. One of these lncRNAs, LINC02568, was found to be highly expressed in ER^+^ breast cancer cell lines and clinical specimen. Functional studies revealed that LINC02568 is essential for the proliferation, migration, and invasion as well as drug resistance of ER^+^ breast cancer cells. Mechanically, we demonstrated that LINC02568 regulates estrogen/ER*α*‐induced gene transcriptional activation *in trans* by stabilizing *ESR1* mRNA in the cytoplasm. Meanwhile, LINC02568 contributes to tumor‐specific pH homeostasis by regulating carbonic anhydrase CA12 *in cis* in the nucleus. ASO specifically targeting LINC02568 can significantly attenuate ER^+^ breast cancer cell growth in vitro and tumor growth in vivo. The combination treatment with ASO targeting LINC02568 and endocrine therapy drugs or CA inhibitor achieved better therapeutic effects.

## 2 Results

### 2.1 Estrogen Induces the Expression of a Large Number of mRNAs as well as lncRNAs Nearby in ER^+^ Breast Cancer Cells

**Figure 1:**
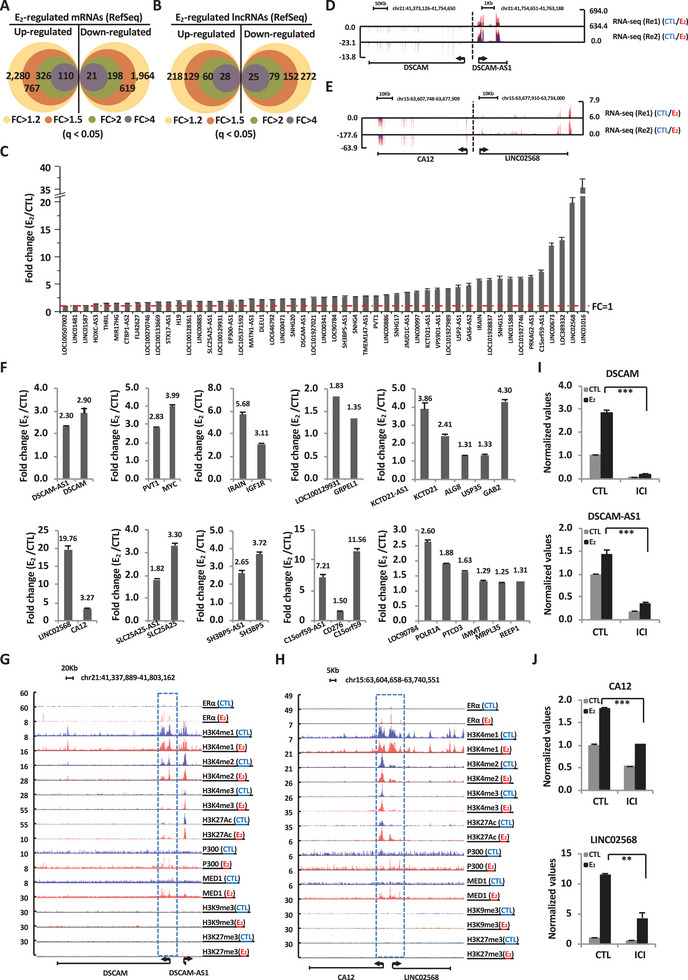
Estrogen induces the expression of a large cohort of mRNAs as well as lncRNAs nearby in ER^+^ breast cancer cells. A,B) MCF7 cells were treated with estrogen (E_2_, 10^−7^ m, 6 h) followed by RNA extraction and RNA‐seq analysis. Venn diagram shows the number of mRNAs (A) and lncRNAs (B) annotated in RefSeq that were regulated, both up‐ and downregulated, by estrogen under different cutoff (*q* < 0.05). C) RNA sample as described in (A) were subjected to RT‐qPCR analysis to examine the expression of representative, estrogen‐induced lncRNAs as detected by RNA‐seq analysis (±SEM). D,E) UCSC genome browser views of RNA‐seq for estrogen‐induced lncRNAs and coding genes nearby as indicated are shown. F) RNA samples as described in (A) were subjected to RT‐qPCR analysis to examine the expression of estrogen‐induced lncRNAs and coding genes nearby as indicated (±SEM). G,H) UCSC genome browser views of ER*α*, H3K4me1, H3K4me2, H3K4me3, H3K27Ac, P300, H3K9me3, and H3K27me3 ChIP‐seq tag density in the presence (red) or absence (blue) of estrogen on genomic regions for estrogen‐induced lncRNAs and coding genes nearby as indicated. I,J) MCF7 cells were treated with or without fulvestrant (ICI, 1 × 10^−6^ m, 6 h) in the presence or absence of estrogen (E_2_, 10^−7^ m, 6 h) followed by RNA extraction and RT‐qPCR analysis to examine the expression of lncRNAs and coding genes nearby as indicated (±SEM, ^***^ *p* < 0.001, ^**^ *p* < 0.01).

To explore key regulators of estrogen/ER*α* signaling pathway in ER^+^ breast cancer, we profiled estrogen‐regulated transcriptome in MCF7 cells (Figure S1A, Supporting Information). In total, 767 and 619 mRNAs were found to be induced and repressed by estrogen, respectively (*q* < 0.05, FC > 1.5) (**Figure** **1**A). Meanwhile, a large cohort of lncRNAs were found to be regulated by estrogen (Figure 1B). The induction of representative lncRNAs by estrogen was well validated by RT‐qPCR analysis (Figure 1C). We also examined estrogen‐regulated lncRNAs in other ER^+^ breast cancer cells, including CAMA‐1, EFM‐19, HCC1500, MDA‐MB‐134‐VI, and T‐47D, based on RNA‐seq reported previously.^[^ ^42^ ^]^ There were 135 and 106 lncRNAs being induced and repressed, respectively, in at least three cell lines (Figure S1B and Table S1, Supporting Information).

It has been reported that lncRNAs are often co‐induced with nearby coding genes.^[^ ^43^ ^]^ We therefore analyzed the coexpression pattern of estrogen‐induced lncRNAs and their nearby coding genes in MCF7 cells. Of all estrogen‐induced lncRNAs, 63.27% of them were co‐induced with their nearby mRNAs. The co‐induction of representative lncRNA‐mRNA pairs/hubs was shown in UCSC genome browser viewer and further validated by RT‐qPCR analysis (Figure 1D–F). The expression of lncRNA‐mRNA pairs was also examined in a cohort of normal and ER^+^ breast tumor tissues (*n* = 10). The results showed that C15orf59‐AS1/C15orf59 were simultaneously upregulated in all ER^+^ breast tumors, and the rest of lncRNA/mRNA pairs were upregulated in at least half of the ER^+^ breast tumors examined (Figure S1C, Supporting Information). To test whether these lncRNA‐mRNA pairs/hubs are directly associated with ER*α*, we analyzed the chromatin binding of ER*α* in the presence or absence of estrogen. Around 73% of the lncRNA‐mRNA pairs/hubs had ER*α* binding nearby. These ER*α* binding sites were enriched with active enhancer markers, including H3K4me1/2, H3K27Ac, p300, and MED1, while repressive histone markers, either H3K9me3 or H3K27me3, were found to be absent (Figure 1G,H). The regulation of estrogen‐induced lncRNA‐mRNA pairs by ER*α* was further demonstrated such that treatment with fulvestrant (ICI), the SERD, led to the decreased expression of lncRNAs, such as DSCAM‐AS1 and LINC02568, as well as their mRNA counterparts (Figure 1I,J). These data suggested that a large set of lncRNAs are co‐induced with their nearby coding genes, which are directly regulated by ER*α*.

### 2.2 Estrogen‐Induced lncRNA, LINC02568, Promotes the Malignant Behaviors of ER^+^ Breast Cancer Cells Both In Vitro and In Vivo

**Figure 2:**
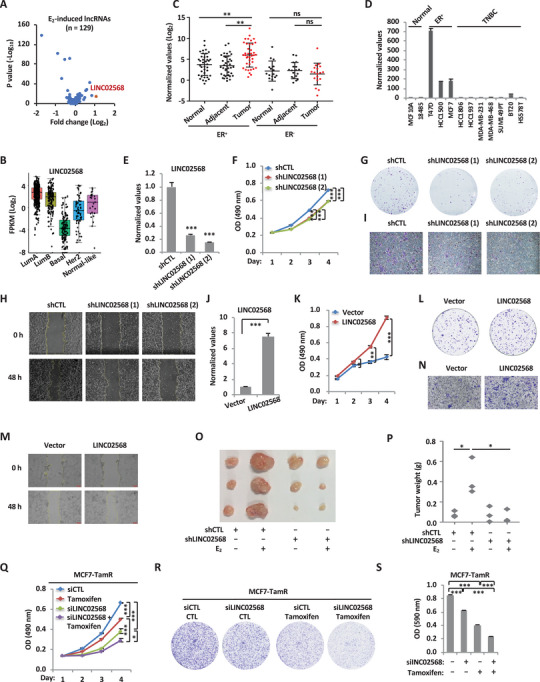
LINC02568 promotes the malignant behaviors of ER^+^ breast cancer cell both in vitro and in vivo. A) Scatter diagram shows the estrogen‐induced lncRNAs detected by RNA‐seq analysis as shown in Figure 1B (FC ≥ 1.5, *n* = 129) that are significantly higher in ER^+^ breast tumor than normal tissues (FC ≥ 2, *p* ≤ 0.01). Red dot represents LINC02568. B) The expression of LINC02568 in different subtypes of breast tumor samples in TCGA portal database is shown (http://tumorsurvival.org/). C) Breast tumors and the corresponding adjacent and normal tissues were collected from breast cancer patients (ER^+^, *n* = 39; ER^−^, *n* = 18) and subjected to RNA extraction and RT‐qPCR analysis to examine the expression of LINC02568 (± s.d., ^**^ *p* < 0.01, ns: nonsignificant). D) Different breast cancer cell lines were collected, followed by RNA extraction and RT‐qPCR analysis to examine the expression of LINC02568 as indicated (±SEM). E) MCF7 cells were infected with lenti‐virus expressing control shRNA (shCTL) or two independent shRNAs specifically targeting LINC02568 (shLINC02568 (1) and shLINC02568 (2)) for 48 h, followed by RNA extraction and RT‐qPCR analysis to examine the expression of LINC02568 (±SEM, ^***^ *p* < 0.001). F–I) MCF7 cells were infected with lenti‐virus expressing shCTL or shLINC02568, and maintained in stripping medium for 48 h before treating with estrogen (E_2_, 10^−7^ m) for duration as indicated, followed by cell proliferation assay (F), colony formation assay (G), wound‐healing assay (H), and Transwell assay (I) (±SEM, ^***^ *p* < 0.001). J) MCF7 cells were infected with control vector or vector expressing LINC02568 for 48 h, followed by RNA extraction and RT‑qPCR analysis to examine the expression of LINC02568 (±SEM, ^***^ *p* < 0.001). K–N) MCF7 cells were infected with control vector or vector expressing LINC02568 and maintained in stripping medium for 48 h before treated with estrogen (E_2_, 10^−7^ m) for duration as indicated, followed by cell proliferation assay (K), colony formation assay (L), wound‐healing assay (M), and Transwell assay (N) (±SEM, ^*^ *p* < 0.05, ^**^ *p* < 0.01, ^***^ *p* < 0.001). O) MCF7 cells infected with lenti‐viral shCTL or shLINC02568 for 48 h were injected subcutaneously into female BALB/C nude mice (*n* = 3), and brushed with or without estrogen (E_2_, 10^−2^ m) on the neck every 2 days. Tumors were collected and photographed. P) The weight of tumors in (O) is shown (± s.d., ^*^ *p* < 0.05). Q,R) Tamoxifen‐resistant MCF7 cells (MCF7‐TamR) were transfected with control siRNA (siCTL) or siRNA specifically targeting LINC02568 (siLINC02568) and treated with tamoxifen (5 µm) for duration as indicated, followed by cell proliferation assay (Q) and colony formation assay (R) (±SEM, ^*^ *p* < 0.05, ^***^ *p* < 0.01, ^***^ *p* < 0.001). S) Quantification of the crystal violet dye in (R) is shown (±SEM, ^***^ *p* < 0.001).

To explore the clinical relevance of estrogen‐induced lncRNAs identified above in MCF7 cells (*q* < 0.05, FC ≥ 1.5, *n* = 129), the expression of these lncRNAs was interrogated into GEPIA2 database. LINC02568 was found to be the most upregulated among lncRNAs that are significantly higher in ER^+^ breast tumor than normal tissues (*p* ≤ 0.01) (**Figure** **2**Α and Table S2, Supporting Information). Further analysis based on TCGA datasets also revealed that LINC02568 is highly expressed in ER^+^ breast tumor tissues (LumA and LumB) compared to other subtypes (Figure 2B). The significantly higher expression of LINC02568 was independently confirmed in a cohort of in‐house ER^+^ breast tumor tissues compared to adjacent or normal tissues as well as tissues from ER‐negative (ER^−^) breast tumor tissues (Figure 2C). Furthermore, LINC02568 is highly expressed in ER^+^ breast cancer cell lines compared with normal mammary epithelial cell lines and other subtypes of breast cancer cell lines, such as triple‐negative breast cancer (Figure 2D). More interestingly, compared to corresponding normal tissues, LINC02568 appears to be only expressed significantly higher in breast tumor tissues, but not other tumor types (Figure S2A, Supporting Information).

The high expression of LINC02568 in clinical breast specimen prompted us to investigate whether it is functional important in ER^+^ breast cancer cell growth and tumorigenesis. To this end, two independent shRNAs were designed to interrupt the expression of LINC02568 (Figure 2E). Knockdown of LINC02568 significantly decreased the proliferation rate of MCF7 cells (Figure 2F) and led to much fewer colonies in colony formation assay (Figure 2G and Figure S2B, Supporting Information). Furthermore, LINC02568 was found to be required for the migration and invasion of MCF7 cells as examined by wound‐healing and Transwell assays, respectively (Figure 2H,I, and Figure S2C,D, Supporting Information). In contrast, overexpression of LINC02568 significantly promoted cell proliferation, colony formation, migration, and invasion ability of MCF7 cells, strengthening the oncogenic role of LINC02568 (Figure 2J–N, and Figure S2E–G, Supporting Information). The requirement of LINC02568 for MCF7 cell proliferation, colony formation, migration, and invasion were confirmed through siRNA specifically targeting LINC02568 (Figure S2H–O), which was further demonstrated in another ER^+^ breast cancer cell line, T47D (Figure S2P–V, Supporting Information). To explore whether LINC02568 is critical for estrogen‐induced tumorigenesis in vivo, BALB/C nude mice were injected subcutaneously with MCF7 cells stably transfected with control shRNA or shRNA targeting LINC02568, and treated with or without estrogen. As expected, LINC02568 knockdown significantly diminished the effects of estrogen‐induced tumor growth (Figure 2O,P). We also investigated the potential role of LINC02568 in overcoming tamoxifen resistance in tamoxifen‐resistant MCF7 cells (MCF7‐TamR). Knockdown of LINC02568 restored the sensitivity of MCF7‐TamR cells to tamoxifen as seen from both cell proliferation and colony formation assays (Figure 2Q–S). Taken together, our data demonstrated that LINC02568 promotes the malignant behaviors as well as endocrine therapy drug resistance, such as tamoxifen, in ER^+^ breast cancer cells.

### 2.3 LINC02568 Regulates the Expression of ER*α In Trans* to Promote the Activation of ER*α*‐Target Genes and the Malignant Behaviors of ER^+^ Breast Cancer

**Figure 3:**
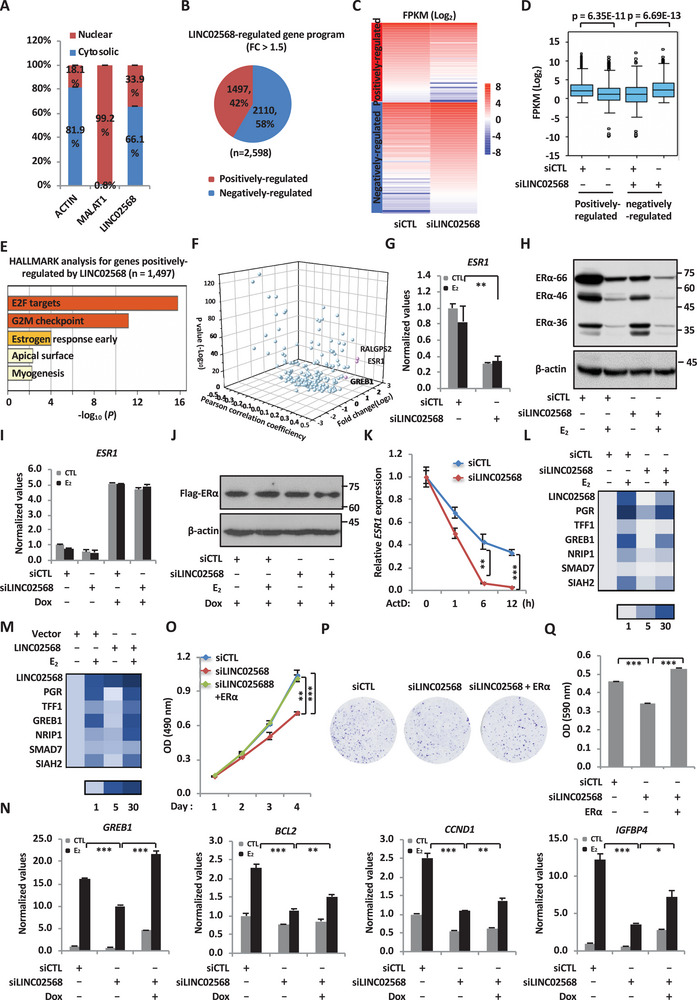
LINC02568 regulates the expression of *ESR1* in trans to promote the malignant behaviors of ER^+^ breast cancer cell. A) MCF7 cells were subjected to cellular fractionation followed by RNA extraction and RT‐qPCR analysis to quantify RNAs as indicated. ACTIN and MALAT1 are served as markers for cytosolic and nuclear faction, respectively. B) MCF7 cells transfected with siCTL or siLINC02568 for 48 h were subjected to RNA‐seq analysis. Genes positively‐ and negatively regulated by LINC02568 are shown by pie chart (FC > 1.5). C,D) Heat map (C) and box plot (D) representation of the expression levels (FPKM, log_2_) for genes regulated by LINC02568 as described in (B). E) Hallmark analysis for genes positively regulated by LINC02568 as described in (B) is shown. F) LINC02568 positively regulated genes that were included in the ceRNA network as shown in Figure S3A (Supporting Information) (*n* = 206) were explored in GEPIA2, and the ones that express significantly higher in ER^+^ breast tumor than normal tissues (FC ≥ 2, *p* ≤ 0.01) and are positively correlated with LINC02568 (*R* ≥ 0.3) are shown in pink dots. G,H) MCF7 cells were transfected with siCTL or siLINC02568 and maintained in stripping medium for 48 h before treated with estrogen (E_2_, 10^−7^ m, 6 h for RT‐qPCR and 24 h for immunoblotting), followed by RNA extraction and RT‐qPCR analysis to examine the expression of *ESR1* (G) and immunoblotting analysis using antibodies as indicated (H). ER*α*−66, ER*α*−46, and ER*α*−36 represent the three ER*α* variants (±SEM, ^**^ *p* < 0.01). I,J) Inducible MCF7 cells stably expressing Flag‐tagged ER*α* were transfected with siCTL or siLINC02568 and maintained in stripping medium with doxycycline (Dox, 1 µg mL^−1^) for 48 h, followed by adding estrogen (E_2_, 10^−7^ m, 6 h for RT‐qPCR and 24 h for immunoblotting) before RT‐qPCR analysis to examine the expression of *ESR1* (I) and immunoblotting analysis using antibodies as indicated (J). K) MCF7 cells transfected with siCTL or siLINC02568 for 48 h were treated with actinomycin D (ActD, 2 µg mL^−1^) for 0, 1, 6, or 12 h as indicated, followed by RNA extraction and RT‐qPCR analysis to examine the expression of *ESR1* (±SEM, ^**^ *p* < 0.01, ^***^ *p* < 0.001). L) RNA samples as described in (G) were subjected to RT‐qPCR analysis to examine the expression of ER*α*‐target genes as indicated (±SEM). M) MCF7 cells were infected with lenti‐virus expressing control vector or LINC02568 and maintained in stripping medium for 48 h before treated with estrogen (E_2_, 10^−7^ m, 6 h). Cells were subjected to RNA exaction and RT‐qPCR analysis to examine the expression of ER*α*‐target genes as indicated (±SEM). N) ER*α*‐inducible MCF7 cells as described in (I) were subjected to RNA extraction and RT‐qPCR analysis to examine the expression of ER*α*‐target genes as indicated (±SEM, ^*^ *p* < 0.05, ^**^ *p* < 0.01, ^***^ *p* < 0.001). O,P) MCF7 cells transfected with siCTL or siLINC02568 in the presence or absence of control vector or vector expressing ER*α* were treated with or without estrogen (E_2_, 10^−7^ m) for duration as indicated, followed by cell proliferation assay (O) and colony formation assay (P). Q) Quantification of the crystal violet dye in (P) is shown (±SEM, ^***^ *p* < 0.001).

To understand the molecular mechanisms underlying LINC02568 regulation of the malignant phenotypes of ER^+^ breast cancer cells, we first examined the coding potential of LINC02568 through CPAT (http://lilab.research.bcm.edu/cpat/). CPAT analysis results showed that LINC02568 has no coding ability (coding probability = 0.0183; coding label: no). We then examined the subcellular localization of LINC02568 by performing cellular fractionation followed by RT‐qPCR analysis. LINC02568 was found to localize in both the cytoplasm and nucleus, but mainly in the cytoplasm in MCF7 cells (**Figure** **3**Α). To gain insights into the molecular mechanisms underlying the oncogenic function of LINC02568, RNA‐seq analysis was performed in MCF7 cells transfected with control siRNA or siRNA targeting LINC02568. There were 1497 and 2110 genes induced and repressed by LINC02568, respectively (*q* < 0.05, FC > 1.5) (Figure 3B). The expression of genes regulated by LINC02568 was shown by heat map and box plot (Figure 3C,D). Hallmark analysis results revealed that E2F targets, G2/M checkpoint, and estrogen response early were the top three most enriched hallmarks for genes positively regulated by LINC02568 (Figure 3E). In particular, the expression of a large number of estrogen‐target genes, such as *PGR*, *TFF1*, *GREB1*, *NRIP1*, *SMAD7*, *SIAH2*, *CCND1*, and *BCL2* was dependent on LINC02568 (Figure 3E).

We next sought to illustrate the molecular mechanisms through which LINC02568 regulates the transcriptional activation of these estrogen‐target genes. As LINC02568 is mainly localized in the cytoplasm, we explored whether LINC02568 could function as a miRNA sponge to regulate target mRNAs. To this end, the competitive endogenous RNA (ceRNA) network was constructed to connect LINC02568 with target genes that are positively regulated by LINC02568 via miRNAs (Figure S3A, Supporting Information). There were 206 genes that are positively regulated by LINC02568 and 5 miRNAs, miR‐1226‐5p, miR‐8070, miR‐6778‐5p, miR‐1233‐5p, and miR‐6849‐3p, in the ceRNA network (Figure S3A, Supporting Information). To identify target genes that are clinically relevant in ER^+^ breast cancer, and also highly correlated with LINC02568, we first queried the expression of those 206 genes in GEPIA2 database, which led to the identification of 18 genes that are expressed significantly higher in ER^+^ breast tumor tissues than normal tissues (FC ≥ 2, *p* ≤ 0.01) (Figure 3F and Table S3, Supporting Information). We then examined the correlation between the expression of LINC02568 and these 18 genes, which led to the identification of three genes, *ESR1*, *RALGPS2*, and *GREB1*, that are highly correlated with LINC02568 in breast tumor tissues (*R* ≥ 0.3) (Figure 3F and Table S4, Supporting Information).

Since the protein product of *ESR1*, ER*α*, is the main driver of estrogen‐target gene transcriptional activation, ER^+^ breast cancer development, and endocrine drug resistance, we proposed that LINC02568 might regulate the expression of *ESR1* to exert its functions in such aspects. We first confirmed that LINC02568 knockdown led to a significant decrease of the mRNA and protein levels of ER*α* (ER*α*−66) (Figure 3G,H). LINC02568 can also regulate the protein level of ER*α* variant, ER*α*−46, which shares the same 3′UTR region as ER*α*−66. Another ER*α* variant, ER*α*−36, which varies in 3′UTR from ER*α*−66 and ER*α*−46, was found to be unaffected (Figure 3H). The downregulation of *ESR1* from RNA‐seq was also shown in UCSC genome browser viewer (Figure S3B, Supporting Information). The effect of LINC02568 on the mRNA and protein levels of ER*α* were also examined in an inducible MCF7 cell line stably expressing ER*α*. LINC02568 showed no obviously effects on the expression of exogenous ER*α*, either mRNA or protein level, suggesting that LINC02568 does not regulate ER*α* at translational stage (Figure 3I,J). The stability of *ESR1* mRNA was found to be decreased upon LINC02568 knockdown (Figure 3K). In consistent with its role in regulating ER*α* expression, knockdown of LINC02568 attenuated the transcriptional activation of ER*α*‐target genes, such as *PGR*, *TFF1*, *GREB1*, *NRIP1*, *SMAD7*, and *SIAH2* (Figure 3L and Figure S3C, Supporting Information). Similar observations were made in T47D cells (Figure S3D, Supporting Information). In contrast, overexpression of LINC02568 further induced the expression of ER*α*‐target genes (Figure 3M).

To examine whether LINC02568 exerts its oncogenic role through regulating ER*α*, ER*α* was reintroduced into MCF7 cells when LINC02568 was knocked down. Reintroducing ER*α* largely rescued the defects in the activation of ER*α*‐target genes when LINC02568 was knocked down (Figure 3N). As expected, the effects of LINC02568 knockdown on cell proliferation and colony formation were also largely rescued by overexpressed ER*α* (Figure 3O–Q). Taken together, our data indicated that LINC02568 regulates ER*α*‐target gene activation and ER^+^ breast cancer cell growth through regulating the expression of *ESR1*.

### 2.4 LINC02568 Serves as a miRNA Sponge of miR‐1233‐5p to Promote the Expression of *ESR1* and ER*α*‐Target Genes, and the Malignant Behaviors of ER^+^ Breast Cancer Cells

**Figure 4:**
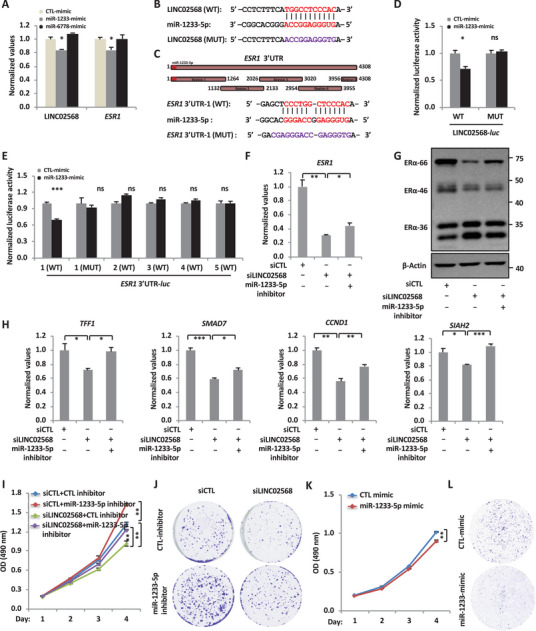
LINC02568 acts as a miRNA sponge for miR‑1233‑5p to regulate the expression of *ESR1* and the malignant phenotypes of ER^+^ breast cancer cell. A) MCF7 cells were transfected with control (CTL), miR‐1233‐5p, or miR‐6778‐5p mimic for 48 h and subjected to RNA extraction and RT‐qPCR analysis to examine the expression of LINC02568 and *ESR1* (±SEM, ^*^ *p* < 0.05). B) Sequence match between miR‐1233‐5p and wild‐type LINC02568 (WT) or corresponding mutant form with the predicted miR‑1233‑5p binding site mutated (MUT) is shown. C) 3′ untranslated region (UTR) of *ESR1* mRNA was divided into five segments (region 1 to 5). Sequence match between miR‐1233‐5p and region 1 of the 3′UTR of *ESR1* (1 (WT)) or the corresponding mutant form with the predicted miR‑1233‑5p binding site mutated (1 (MUT)) is shown. D) MCF7 cells were transfected with luciferase reporter vectors containing wild‑type LINC02568 (WT) or mutant form with the predicted miR‑1233‑5p binding site mutated (MUT) in the presence or absence of control or miR‑1233‑5p mimic for 48 h followed by dual‑luciferase reporter assay (±SEM, ^*^ *p* < 0.05, ns: nonsignificant). E) MCF7 cells were transfected with luciferase reporter vectors containing wild‑type segments from the 3′UTR of *ESR1* (1 (WT), 2 (WT), 3 (WT), 4 (WT), and 5 (WT)) or the mutant form of region 1 with the predicted miR‑1233‑5p binding site mutated (1 (MUT)) in the presence or absence of control or miR‑1233‑5p mimic for 48 h followed by dual‑luciferase reporter assay (±SEM, ^***^ *p* < 0.001, ns: nonsignificant). F–J) MCF7 cells were transfected with siCTL or siLINC02568 in the presence of control or miR‑1233‑5p inhibitor for 48 h, followed by RNA extraction and RT‐qPCR analysis to examine the expression of *ESR1* (F), immunoblotting analysis (G), ER*α*‐target genes (H), cell proliferation assay (I), and colony formation assay (J) (±SEM, ^*^ *p* < 0.05, ^**^ *p* < 0.01, ^***^ *p* < 0.001). K,L) MCF7 cells were transfected with control or miR‑1233‑5p mimic, followed by cell proliferation (K) and colony formation assays (L) (±SEM, ^**^ *p* < 0.01).

We next sought to investigate how LINC02568 regulates the expression of *ESR1*. As revealed by the ceRNA network, LINC02568 was predicted to bind with miR‐1233‐5p and miR‐6778‐5p, which also bind to *ESR1* mRNA (Figure S3A, Supporting Information). To test the effects of these two miRNAs on the expression of LINC02568 and *ESR1*, MCF7 cells were transfected with control, miR‐1233‐5p, or miR‐6778‐5p mimics followed by RT‐qPCR analysis. The overexpression of miR‐1233‐5p significantly reduced the mRNA levels of both LINC02568 and *ESR1*, whereas miR‐6778‐5p showed no significant effects (**Figure** **4**A). We next examined whether miR‐1233‐5p can bind to LINC02568 and the 3′UTR of *ESR1*. The potential binding sites for miR‐1233‐5p in LINC02568 and the 3′UTR of *ESR1* were predicted (Figure 4B,C). Luciferase vectors containing LINC02568, either wild‐type (LINC02568 (WT)‐*luc*) or mutated form with the predicted miR‐1233‐5p binding site mutated (LINC02568 (MUT)‐*luc*), were constructed and then co‐transfect with control mimic or miR‐1233‐5p mimic into MCF7 cells followed by dual‐luciferase assay (Figure 4B,C). The results showed that miR‐1233‐5p mimic transfection significantly reduced the luciferase activity of LINC02568 (WT)‐*luc*, but not LINC02568 (MUT)‐*luc* (Figure 4D). Similarly, the miR‐1233‐5p mimic specifically reduced the luciferase activity of *ESR1* 3′UTR1‐*luc*, which contains the miR‐1233‐5p binding site, but not others including *ESR1* 3′UTR2‐*luc*, *ESR1* 3′UTR3‐*luc*, *ESR1* 3′UTR4‐*luc*, or *ESR1* 3′UTR5‐*luc*, which has no miR‐1233‐5p binding site predicted (Figure 4E). *ESR1* 3′UTR1 (MUT)‐*luc* also showed no responses to miR‐1233‐5p mimic (Figure 4E). These data suggested that miR‐1233‐5p binds to both LINC02568 and *ESR1* mRNA, and regulates their expression.

Next, we tested whether LINC02568 regulation of *ESR1* and ER*α*‐target genes is through miR‐1233‐5p. MCF7 cells were transfected with control siRNA or siRNA targeting LINC02568 in the presence or absence of miR‐1233‐5p inhibitor followed by RT‐qPCR analysis to examine the expression of *ESR1* and ER*α*‐target genes. The results showed that the inhibition of miR‐1233‐5p partially rescued the expression of *ESR1* (Figure 4F,G) as well as ER*α*‐target genes (Figure 4H). To examine whether LINC02568 regulation of the malignant behaviors of ER^+^ breast cancer cells is through miR‐1233‐5p, we first examined the effects of miR‐1233‐5p itself. The expression of miR‐1233‐5p showed no significant changes in ER^+^ breast tumor compared to normal tissues (Figure S3E, Supporting Information). No significant changes of miR‐1233‐5p were observed in LINC02568‐knockdown tumors as described in Figure 2O (Figure S3F, Supporting Information). MiR‐1233‐5p loss led to a significant increase in the cell proliferation rate and colony numbers in MCF7 cells, while overexpression of miR‐1233‐5p exhibited the opposite effects, indicating that miR‐1233‐5p plays a tumor suppressor role (Figure 4I–L, and Figure S3G,H, Supporting Information). Consistently, the defects in cell proliferation and colony formation caused by LINC02568 knockdown were partially rescued by inhibition of miR‐1233‐5p in MCF7 cells (Figure 4I,J, and Figure S3G, Supporting Information). Taken together, our data suggested that LINC02568 promotes the activation of ER*α*‐target genes and ER^+^ breast cancer cell growth by regulating the expression of *ESR1* through sponging miR‐1233‐5p.

### 2.5 LINC02568 Regulates the Expression of *CA12* *In Cis* to Control Tumor‐specific pH Homeostasis and Promote the Malignant Behaviors of ER^+^ Breast Cancer Cells

**Figure 5:**
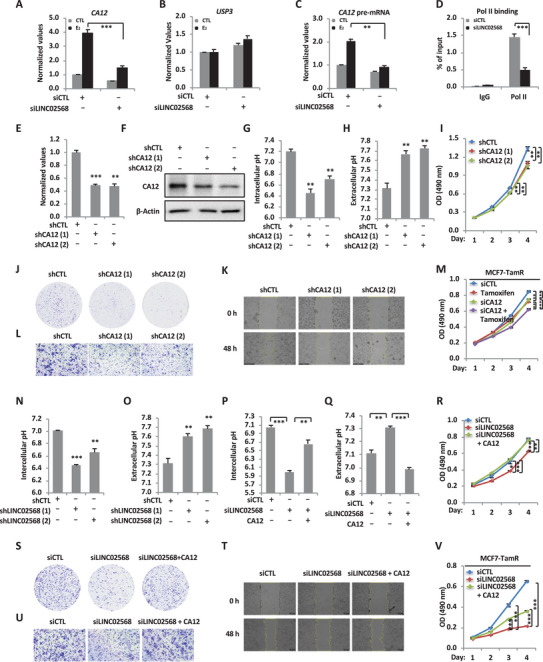
LINC02568 regulates the expression of *CA12* *in cis* to control tumor‐specific pH homeostasis and promote the malignant behaviors of ER^+^ breast cancer cells. A–C) MCF7 cells were transfected with siCTL or siLINC02568 and maintained in stripping medium for 48 h before treated with estrogen (E_2_, 10^−7^ m, 6 h), followed by RNA extraction and RT‐qPCR analysis to examine the expression of *CA12* (A), *USP3* (B), and pre‐mRNA of *CA12* (C) (±SEM, ^**^ *p* < 0.01, ^***^ *p* < 0.001). D) MCF7 cells transfected with siCTL or siLINC02568 for 48 h were subjected to control IgG or RNA polymerase II (pol II) ChIP‐qPCR analysis to examine the binding of RNA pol II on the promoter region of CA12. Data shown is the percentage of input (±SEM, ^***^ *p* < 0.001). E–L) MCF7 cells infected with lenti‐virus expressing control or two independent shRNAs targeting CA12 (shCA12 (1) and shCA12 (2)) for 48 h were subjected to RT‐qPCR (E), immunoblotting analysis (F), intracellular pH measurement (G), extracellular pH measurement (H), cell proliferation assay (I), colony formation assay (J), wound‐healing assay (K), and Transwell assay (L) (±SEM, ^**^ *p* < 0.01, ^***^ *p* < 0.001). M) MCF7‐TamR cells transfected with siCTL or siCA12 and treated with tamoxifen (5 µm) for duration as indicated were subjected to cell proliferation assay (±SEM, ^**^ *p* < 0.01, ^***^ *p* < 0.001). N,O) MCF7 cells infected with lenti‐virus expressing shCTL or shLINC02568 for 48 h were subjected to intracellular (N) or extracellular (O) pH measurement (±SEM, ^**^ *p* < 0.01, ^***^ *p* < 0.001). P–U) MCF7 cells transfected with siCTL or siLINC02568 in the presence of control vector or vector expressing CA12 were subjected to intracellular pH measurement (P), extracellular pH measurement (Q), cell proliferation assay (R), colony formation assay (S), wound‐healing assay (T), and Transwell assay (U) (±SEM, ^*^ *p* < 0.05, ^**^ *p* < 0.01, ^***^ *p* < 0.001). V) MCF7‐TamR cells transfected with siCTL or siLINC02568 in the presence of control vector or vector expressing CA12 and treated with tamoxifen (5 × 10^−6^ m) for duration as indicated were subjected to cell proliferation assay (±SEM, ^***^ *p* < 0.001).

Due to the facts that LINC02568 is localized in both the cytoplasm and nucleus of cells (Figure 3A), and LINC02568 was co‐induced with a divergent gene, CA12, under estrogen stimulation (Figure 1H), we proposed that LINC02568 might function *in cis* in addition to its *in trans* mechanism to exert its oncogenic role in ER^+^ breast cancer. To this end, we first demonstrated that knockdown of LINC02568 attenuated the expression of CA12, but not USP3, another neighboring gene that is not responsive to estrogen treatment (**Figure** **5**A,B,). It has been well demonstrated that divergent lncRNAs are often located in the same “topologically associating domains” (TADs) with nearby genes to regulate the chromatin accessibility of nearby gene promoters and therefore the transcriptional activation of nearby genes.^[^ ^43^, ^44^, ^45^ ^]^ The regulation of CA12 by LINC02568 appeared to occur at the transcriptional stage as the pre‐mRNA levels of CA12 were found to be attenuated when LINC02568 was knocked down (Figure 5C). The mRNA and pre‐mRNA levels of CA12 were also found to be decreased in LINC02568‐knockdown tumors as described in Figure 2O (Figure S4A,B, Supporting Information). To support the transcriptional regulation of CA12 by LINC02568, the recruitment of RNA polymerase II to CA12 promoter region was significantly decreased upon LINC02568 knockdown (Figure 5D). Similar as LINC02568, CA12 was also found to be highly expressed in ER^+^ breast tumors (LumA and LumB) compared to other subtypes based on TCGA database (Figure S4C, Supporting Information). The significantly higher expression of CA12 was independently confirmed in a cohort of in‐house ER^+^ breast tumor tissues compared to adjacent or normal tissues as well as tissues from ER^−^ breast tumor tissues (Figure S4D, Supporting Information). Furthermore, LINC02568 is highly expressed in ER^+^ breast cancer cell lines compared with normal mammary epithelial cells and other subtypes of breast cancer cells, such as triple‐negative breast cancer (Figure S4E, Supporting Information). To further strengthen the regulation of CA12 by LINC02568, the expression of CA12 was found to be the most correlated with that of LINC02568 based on ER^+^ breast tumor tissues as reported in TANRIC database (https://www.tanric.org) (Figure S4F and Table S5, Supporting Information). The high correlation between the expression of LINC02568 and CA12 was independently confirmed in a cohort of ER^+^ breast tumor tissues in‐house (Figure S4G, Supporting Information). CA12 was the most abundant among all members in the CA family and the only one to be induced by estrogen, strengthening the predominant role of CA12 (Figure S4H, Supporting Information). Taken together, our data revealed that LINC02568 regulates nearby *CA12* gene *in cis*, and the expression of both genes is highly correlated in ER^+^ breast cancer.

We next investigated whether regulation of CA12 *in cis* by LINC02568 contributes to its oncogenic role in ER^+^ breast cancer. CA12 belongs to the protein family of membrane‐associated CAs, which have been shown to be involved in creating and maintaining the pH difference between the intra‐ and extracellular spaces in the hypoxic tumor microenvironment, thereby promoting tumor cell growth and metastasis. We first tested whether CA12 has any effects on the pH homeostasis in ER^+^ breast cancer cells. Two individual shRNAs specifically targeting CA12 were designed to knock down the expression of CA12 in MCF7 cells (Figure 5E,F). The intracellular pH of MCF7 cells were significantly reduced after CA12 knockdown (Figure 5G), while the extracellular environment became more alkaline (Figure 5H). We next tested whether CA12 is required for the malignant phenotypes of MCF7 cells. Knockdown of CA12 remarkably inhibited the cell proliferation, colony formation, migration, and invasion ability of MCF7 cells (Figure 5I–L, and Figure S5A–C, Supporting Information). Similar effects on intracellular and extracellular pH, cell proliferation, migration, and invasion were seen when CA12 was knocked down using siRNA approaches (Figure S5D–N, Supporting Information). In contrast, CA12 overexpression exhibited the opposite effects, further strengthening the critical role of CA12 in controlling tumor‐specific pH homeostasis and promoting the malignant phenotypes of MCF7 cells (Figure S5O–W). Furthermore, knockdown of CA12 inhibited cell proliferation in MCF7‐TamR cells (Figure 5M).

Having demonstrated that it is functional important in controlling tumor‐specific pH homeostasis and promoting the malignant behaviors of ER^+^ breast cancer cell, we next tested whether CA12, as a downstream target of LINC02568, contributes to the oncogenic function of LINC02568. First, knockdown of LINC02568 led to a decreased intracellular pH, but increased extracellular pH in MCF7 cells (Figure 5N,O, and Figure S5X,Y, Supporting Information). In contrast, overexpression of LINC02568 resulted in a more acidic extracellular environment (Figure S5Z, Supporting Information). Meanwhile, knockdown of LINC02568 decreased the extracellular acidification rate (ECAR) and the nonglycolytic acidification in MCF7 cells, while overexpression of LINC02568 displayed the opposite effect, indicating that LINC02568 not only participated in the regulation of extracellular acidification in basal conditions, but also participated in the regulation of downstream cellular metabolic activities, such as glycolysis (Figure S5X’–Y’, Supporting Information). Next, to examine whether CA12 contributes to LINC02568 function in cellular pH control and malignant phenotypes of ER^+^ breast cancer cell, we performed rescue experiments in which CA12 was introduced when LINC02568 was knocked down in MCF7 cells. Overexpressed CA12 significantly rescued the effects of LINC02568 on intracellular and extracellular pH, as well as cell proliferation, colony formation, migration, and invasion (Figure 5P–U, and Figure S5Z’–Y’’, Supporting Information). Furthermore, overexpression of CA12 in MCF7‐TamR cells could partially reverse the effect of LINC02568 knockdown on tamoxifen‐sensitization (Figure 5V). Taken together, our data revealed that LINC02568 regulates the expression of CA12 to control the pH homeostasis and promote the malignant phenotypes of ER^+^ breast cancer cells.

### 2.6 LINC02568 Serves as a Potential Therapeutic Target for ER^+^ Breast Cancer

**Figure 6:**
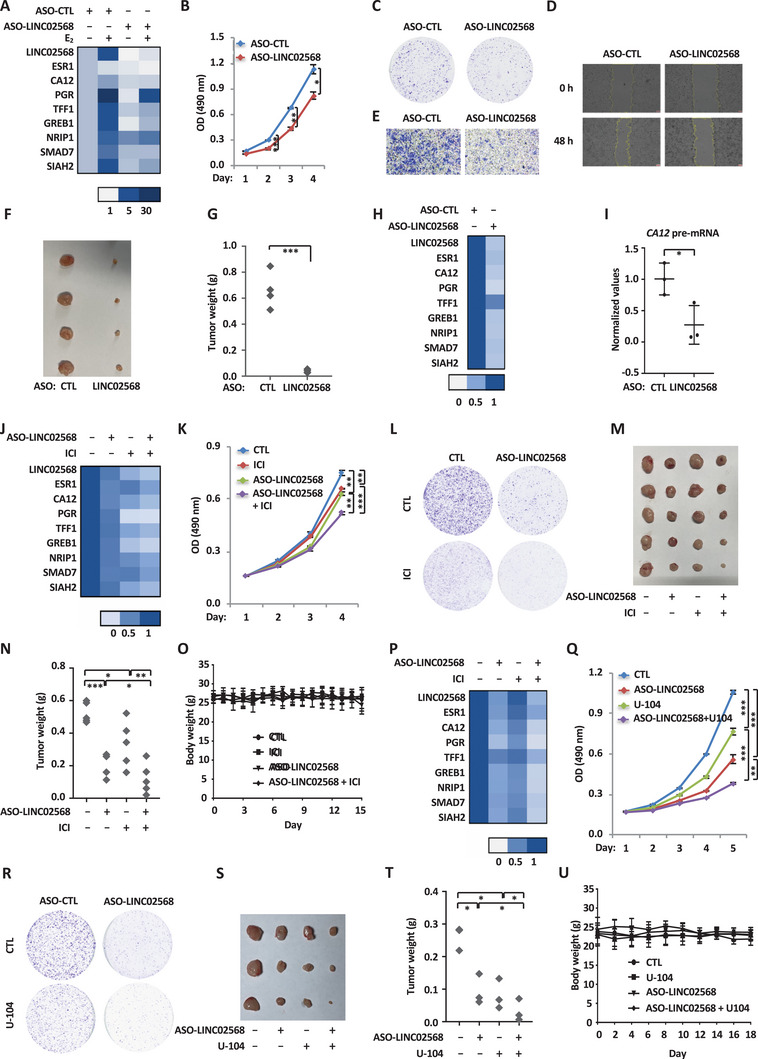
Antisense oligonucleotides (ASO) targeting LINC02568 is potent in suppressing ER^+^ breast tumor growth. A) MCF7 cells were transfected with control ASO (ASO‐CTL) or ASO specially targeting LINC02568 (ASO‐LINC02568) and maintained in stripping medium for 48 h before treating with estrogen (E_2_, 10^−7^ m, 6 h), followed by RNA extraction and RT‐qPCR analysis to examine the expression of LINC02568, *ESR1*, *CA12*, and representative ER*α*‐genes as indicated (±SEM). B–E) MCF7 cells were transfected with ASO‐CTL or ASO‐LINC02568 and maintained in stripping medium for 48 h before treating with estrogen (E_2_, 10^−7^ m) for duration as indicated, followed by cell proliferation assay (B), colony formation assay (C), wound‐healing assay (D), and Transwell assay (E) (±SEM, ^*^ *p* < 0.05, ^**^ *p* < 0.01, ^***^ *p* < 0.001). F) MCF7 cells were injected subcutaneously into female BALB/C nude mice, and brushed with estrogen (E_2_, 10^−2^ m) on the neck every 2 days until tumor size reached approximately 100 mm^3^. Mice were then randomly assigned into two groups (*n* = 4) and injected with ASO intratumorally every 2 days at a dose of 5 nmol per injection (50 µL, 0.1 m in PBS) for eight times. Tumors were harvested, photographed, and weighted. G) The weight of tumors in (F) is shown (± s.d., ^***^ *p* < 0.001). H) Tumors as described in (F) were subjected to RNA extraction and RT‐qPCR analysis to examine the expression of LINC02568, *ESR1*, *CA12*, and representative ER*α*‐target genes as indicated (±SEM). I) Tumors as described in (F) were subjected RNA extraction and RT‐qPCR analysis to examine the expression of pre‐mRNA of *CA12* (± s.d., ^*^ *p* < 0.05). J) MCF7 cells were transfected with ASO‐CTL or ASO‐LINC02568 and then maintained in stripping medium for 48 h before treated with estrogen (E_2_, 10^−7^ m, 6 h) in the presence or absence of fulvestrant (ICI, 1 µm, 6 h) followed by RNA extraction and RT‐qPCR analysis to examine the expression of LINC02568, *ESR1*, *CA12*, and representative ER*α*‐target genes as indicated (±SEM). K,L) MCF7 cells were transfected with ASO‐CTL or ASO‐LINC02568 and treated with or without fulvestrant (ICI, 2.5 µm) for duration as indicated, followed by cell proliferation assay (K) and colony formation assay (L) (±SEM, ^**^ *p* < 0.01, ^***^ *p* < 0.001). M) MCF7 cells were injected subcutaneously into female BALB/C nude mice, and brushed with estrogen (E_2_, 10^−2^ m) on the neck every 2 days until tumor size reached approximately 100 mm^3^. Mice were then randomly assigned into four groups (*n* = 5), and injected intratumorally with ASO every 2 days at a dose of 5 nmol per injection (50 µL, 0.1 m in PBS) for six times, and/or subcutaneously with fulvestrant (ICI) once a week at a dose of 250 mg kg^−1^. Tumors were harvested, photographed, and weighted. N) The weight of tumors in (M) is shown (± s.d., ^**^ *p* < 0.01, ^***^ *p* < 0.001). O) The body weight of mice as described in (M) is shown (± s.d.). P) Tumors as described in (M) were subjected to RNA extraction and RT‐qPCR analysis to examine the expression of LINC02568, *ESR1*, *CA12*, and representative ER*α*‐target genes as indicated (±SEM). Q,R) MCF7 cells were transfected with ASO‐CTL or ASO‐LINC02568 and treated with or without U‐104 (50 µm) for duration as indicated, followed by cell proliferation assay (Q) and colony formation assay (R) (±SEM, ^**^ *p* < 0.01, ^***^ *p* < 0.001). S) MCF7 cells were injected subcutaneously into female BALB/C nude mice, and brushed with estrogen (E_2_, 10^−2^ m) on the neck every 2 days until tumor size reached approximately 100 mm^3^. Mice were then randomly assigned into four groups (*n* = 3), and injected intratumorally with ASO every 2 days at a dose of 5 nmol per injection (50 µL, 0.1 m in PBS) for six times, and/or subcutaneously with U‐104 every other day at a dose of 50 mg kg^−1^. Tumors were harvested, photographed, and weighted. T) The weight of tumors in (S) is shown (±s.d., ^*^ *p* < 0.05). U) The body weight of mice as described in (S) is shown (±s.d.).

The important regulatory functions of LINC02568 for ER*α*‐target gene activation and pH homeostasis, and therefore tumor growth and drug resistance in ER^+^ breast cancer cells prompt us to explore the potential of LINC02568 as a therapeutic target. To this end, ASO specifically targeting LINC02568 was designed and synthesized to interfere with the expression of LINC02568 (**Figure** **6**A). ASO targeting LINC02568 reduced the expression of downstream target genes (Figure 6A). Moreover, the interference of LINC02568 by ASO significantly inhibited cell proliferation, colony formation, migration, and invasion of MCF7 cells (Figure 6B–E, and Figure S6A–C, Supporting Information). To investigate the effect of ASO targeting LINC02568 in vivo, nude mice were subcutaneously inoculated with MCF7 cells and treated with estrogen in the presence of cholesterol‐modified control ASO or ASO targeting LINC02568. ASO targeting LINC02568 injected intratumorally significantly diminished the effects of estrogen‐induced tumor growth (Figure 6F,G). As expected, the expression of LINC02568‐target genes was found to be significantly reduced in LINC02568 ASO‐treated tumors, while the expression of miR‐1233‐5p showed no obvious change (Figure 6H,I, and Figure S6D, Supporting Information). Though it is not as dramatic as intratumoral injection, intravenous injection of ASO targeting LINC02568 also inhibited tumor growth (Figure S6E,F, Supporting Information).

The positive feedback loop between ER*α* and LINC02568 suggested that combination therapy with ASO targeting LINC02568 and endocrine therapy drugs, such as tamoxifen and fulvestrant (ICI), might achieve synergistic effects on tumor inhibition. To test this, MCF7 cells were treated with or without ICI in the presence or absence of ASO targeting LINC02568. Combination treatment with ASO and ICI exhibited much better effects on target gene expression compared to ASO or tamoxifen treatment alone (Figure 6J). Consistently, combination treatment exhibited synergistic effects on cell proliferation and colony formation (Figure 6K,L, and Figure S6G, Supporting Information). Similar effects were also observed when ASO targeting LINC02568 was combined with tamoxifen (Figure S6H–K). We further tested the effects of combination treatment with ICI and ASO on tumor growth in mouse xenograft models. In consistent with what we observed in cultured cells, combination treatment exhibited synergistic effects on tumor inhibition from MCF7 cells (Figure 6M,N), while it had no significant impact on mice body weight (Figure 6O). The expression of LINC02568‐target genes was also found to be inhibited in a synergistic manner in tumor tissues with combination treatment (Figure 6P). We also tested the effects of combination therapy with ASO targeting LINC02568 and U‐104, a small molecule inhibitor capable of inhibiting CA12 activity. Our results showed that combination treatment exhibited synergistic effects on cell proliferation and colony formation in MCF7 cells (Figure 6Q,R, and Figure S6L, Supporting Information), as well as MCF7 cells‐derived tumor growth in mouse (Figure 6S–U). Similar as ASO targeting LINC02568, U‐104 treatment made MCF7 cells more sensitive to tamoxifen treatment as examined by cell proliferation and colony formation assays (Figure S6M–O). Taken together, our observation indicated that LINC02568 serves as a potential therapeutic target in ER^+^ breast cancer, such that ASO targeting LINC02568 alone or in combination with endocrine therapy drugs or CA12 inhibitor is potent in inhibiting ER^+^ breast tumor growth.

## 3 Discussion

**Figure 7:**
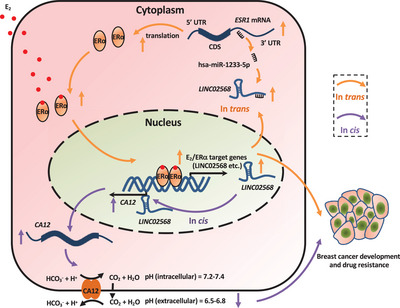
A proposed model of LINC02568 in promoting ER^+^ breast cancer development and drug resistance through dual mechanisms, both *in* *trans* and *in cis*. Estrogen/ER*α*‐induced LINC02568 acts as a ceRNA to sponge miR‐1233‐5p to regulate the expression of *ESR1* in the cytosol of cells, in turn promoting the activation of ER*α*‐target gene program. LINC02568 also regulates its adjacent coding gene *CA12 in cis* to regulate cellular pH. LINC02568‐regulated ER*α*‐target gene activation and cellular pH both contribute to the development and drug resistance of ER^+^ breast cancer.

The aberrant activation of estrogen/ER*α*‐mediated signaling is the one of the major drivers of ER^+^ breast cancer. By activating the transcription of enormous genes, ER*α* participates in the regulation of various functions in ER^+^ breast tumor cells. Endocrine therapy is the frontline treatment for ER^+^ breast patients. However, primary and acquired resistance to endocrine therapy remains as a big challenge in clinic. Except for targeting ER*α* itself, inhibition of the key regulators in the estrogen signaling pathway is also a promising therapeutic strategy for ER^+^ breast cancer. Previous studies have demonstrated that a large amount of lncRNAs can be induced by estrogen. However, the characterization, functions, and mechanisms of these lncRNAs have not been fully elucidated yet.^[^ ^16^, ^46^, ^47^, ^48^, ^49^ ^]^ In this study, we identified an estrogen‐induced lncRNA, LINC02568, which is clinically relevant in ER^+^ breast cancer and functionally important for the malignant phenotypes and tamoxifen resistance in ER^+^ breast cancer cells. Our results revealed a positive feedback loop between LINC02568 and ER*α* that amplifies the estrogen signaling in ER^+^ breast cancer, in which estrogen/ER*α* activates the expression of LINC02568, and LINC02568 in turn regulates the expression of ER*α* through a ceRNA mechanism to boost estrogen/ER*α*‐mediated gene transcriptional program (**Figure** **7**).

The change between intracellular (pHi) and extracellular pH (pHe) in cells is a hallmark of tumor. In most normal cells, the pHe is maintained at a slightly alkaline condition (≥7.3), while the pHi is approximately 7.2. However, due to the increased lactate production and excretion, the pHe is reduced to 6.5–7.1, while the pHi becomes slightly alkaline (≥7.2) in tumor cells, which is beneficial for tumor cell growth and metastasis.^[^ ^29^, ^30^, ^31^ ^]^ The altered expression of some key proteins is proved to be responsible for the pH change in tumor cells, including Na^+^/H^+^ exchanger 1 (NHE1), Vacuolar H^+^ ATPases (V‐ATPases), and CAs.^[^ ^29^ ^]^ We demonstrated here that CA12, a member in the CA protein family, is critical for maintaining the pH homeostasis and promoting the malignant phenotypes of ER^+^ breast cancer cells. It has been reported that lncRNAs can function *in cis* to regulate the expression of their neighboring coding genes.^[^ ^43^, ^45^, ^50^ ^]^ LINC02568 appears to play an important role in pH homeostasis maintenance through its *cis*‐regulatory effects of LINC02568. In breast tumor cells, the high expression of LINC02568 maintains the high transcriptional activity of CA12, and therefore pH homeostasis. The mechanisms of *cis*‐acting lncRNAs in activating target coding genes can result from either lncRNAs transcripts themselves, the transcription of lncRNAs, or even the DNA elements in the lncRNAs loci.^[^ ^50^, ^51^, ^52^, ^53^, ^54^ ^]^ The most general feature of lncRNAs is taking the form of R‐loops (triple‐stranded nucleic acid structures with RNA hybridized to duplex DNA) with DNA and proteins to influence chromatin accessibility and thereby mediating neighboring gene activation or silencing.^[^ ^44^, ^45^, ^55^, ^56^, ^57^, ^58^ ^]^ The existence of this regulatory mechanism allows low levels of lncRNA copies in the nucleus to affect the ubiquitous expression of adjacent genes, as only two DNA copies are present per cell.^[^ ^51^ ^]^ Here, given the fact that knockdown of LINC02568 significantly attenuates the transcription of CA12, and the low levels of LINC02568 in the nucleus of cells, whether LINC02568 regulates CA12 by forming R‐loop warrants future investigation. It is worth noting that CA12 is also one of the target genes of ER*α*. Therefore, LINC02568 might regulate the expression of CA12 through dual mechanisms, both *in trans* and *in cis*.

The functional importance of LINC02568 in regulating estrogen/ER*α*‐mediated gene transcriptional activation and pH homeostasis, and therefore the malignant behaviors of ER^+^ breast cancer indicates that it has great potential as a therapeutic target. ASO drugs are considered to be a very promising RNA‐targeted intervention since they have shown high‐efficacy and long‐term action in vivo. ASO specifically targeting LINC02568 significantly inhibits ER^+^ breast tumor growth, and combination therapy with endocrine therapy drug, such as tamoxifen and ICI, exhibited synergistic effects. More importantly, due to the involvement of ER*α*‐target genes and CA12 in endocrine therapy drug resistance, the combination of ASO targeting LINC02568 and tamoxifen can restore the sensitivity of tamoxifen‐resistant cells to tamoxifen. LINC02568 therefore serves as a potential therapeutic target in ER^+^ breast cancer, and ASO targeting LINC02568 might provide a promising treatment option for ER^+^ breast cancer patients with primary and acquired endocrine therapy drug resistance.

## 4 Experimental Section

### Clinical Specimens and Cell Lines

Breast tumor tissues and matched adjacent normal tissues were obtained from patients who were diagnosed with breast cancer and who had undergone surgery at The Second Affiliated Hospital of Shantou University Medical College. Tissue samples were freshly frozen in liquid nitrogen and stored at −80 °C until RNA extraction. The study was approved by the Institutional Ethics Committee of the Second Affiliated Hospital of Shantou University Medical College. All research was performed in compliance with government policies and the Helsinki Declaration. Experiments were undertaken with the understanding and written consent of each subject.

Human breast cancer cell lines and human embryonic kidney 293T cells were cultured in Dulbecco's modified Eagle medium (DMEM) (Biological Industries) or Roswell Park Memorial Institute 1640 (RPMI 1640) (Biological Industries). All medium was supplied with 10% fetal bovine serum (FBS) (Biological Industries) and 1% penicillin/streptomycin (Biological Industries). Tamoxifen resistant MCF7 (MCF7‐TamR) cells were maintained in culture medium with tamoxifen (1 µm). When treating cells with estrogen, phenol red‐free medium supplemented with 5% charcoal‐treated FBS was used.

### RNA Isolation and RT‐qPCR

Total RNA was isolated using Trizol (Takara) following the manufacturer's protocol. First‐strand cDNA synthesis from total RNA was carried out using All‐In‐One RT MasterMix (Abm), followed by quantitative PCR (qPCR) using AriaMx Real‐Time (RT) PCR machine (Agilent Technologies). Three biological repeats have been done, and representative data was shown. Standard error of the mean is depicted. Sequence information for all primers used to check gene expression was presented in Table S6 (Supporting Information).

### RNA Sequencing (RNA‐seq) and Computational Analysis of RNA‐seq Data

Total RNA was extracted from MCF7 cells using RNeasy Mini Kit (Qiagen) following the manufacturer's protocol. DNase I in column digestion was included to ensure the RNA quality. RNA library preparation was performed by using NEBNext UltraDirectional RNA Library Prep Kit for Illumina (E7420L). Paired‐end sequencing was performed with Illumina HiSeq 3000 platform. Sequencing reads were aligned to the human reference genome (hg19) by using STAR. Cufflinks was used to calculate the expression of RefSeq annotated genes with the option—M (reads aligned to repetitive regions were masked) and ‐u (multiple aligned read are corrected using “rescue method”). RNA‐seq files were deposited in the Gene Expression Omnibus database under accession GSE217190. The following link has been created to allow review of record GSE217190 while it remains in private status: https://www.ncbi.nlm.nih.gov/geo/query/acc.cgi?acc = GSE217190 (token: kvmfgemclpyxlwr).

### Molecular Cloning

ShRNAs (small hairpin RNAs) targeting LINC02568 and CA12 were cloned into lenti‐viral pLKO.1 vector between AgeI and EcoRI restriction enzyme sites. Sequence information for all shRNAs used was presented in Table S7 (Supporting Information).

Full‐length LINC02568 and CA12 were amplified from MCF7 cells and cloned into the pCDH‐CMV‐MCS‐EF1‐puro expression vector with XbaI and NotI restriction enzymes. Full‐length ESR1 was amplified from MCF7 cells and cloned into the pBoBi‐ expression vector with BamHI and XhoI restriction enzymes for overexpression.

LINC02568 (LINC02568 (WT)) as well as its mutant form with the miR‐1233‐5p binding site mutated (LINC02568 (MUT)) were cloned into psiCHECK2 expression vector with NotI and XhoI restriction enzymes.

Segments from the 3′UTR of *ESR1* (*ESR1*‐3′UTR (WT)) and the mutant form with the miR‐1233‐5p binding site mutated (*ESR1*‐3′UTR (MUT)) were amplified from MCF7 cells and cloned into the psiCHECK2 expression vector. Sequence information for all primers used for molecular cloning was presented in Table S6 (Supporting Information).

### SiRNAs, miRNA Inhibitors, miRNA Mimics, ASOs, and Plasmids Transfection, Lenti‐viral Vectors Packaging and Infection

SiRNAs, miRNA mimics, miRNA inhibitors, and ASOs were all purchased from RiboBio (siCTL (siR NC #1, siN0000001‐1‐5); ASO‐CTL (lncRNA ASO Negative Control #1, lnc6N0000001‐1‐10)). Targeting sequences of siRNAs and ASOs were listed in Table S7 (Supporting Information). SiRNAs, ASOs, and miRNAs transfection were performed using Lipofectamine 2000 (Invitrogen) according to the manufacturer's protocol. Plasmid transfections were performed using Polyethyleneimine (PEI, Polysciences) according to the manufacturer's protocol. For lenti‐viral vectors packaging and infection, HEK293T cells were transfected with lenti‐viral vectors together with packaging vectors, pMDL, VSVG and REV, at a ratio of 10:5:3:2 using Polyethyleneimine (PEI, Polysciences) for 48 h according to the manufacturer's protocol. Virus was collected, filtered and added to MCF7 cells in the presence of 10 µg mL^−1^ polybrene (Sigma, H9268).

### Immunoblotting Analysis

Immunoblotting analysis was performed as described previously.^[^ ^59^ ^]^ Anti‐ER*α* (F‐10) (sc‐8002) was purchased from Santa Cruz Biotechnology; anti‐Flag (F1804) antibody was purchased from Sigma; anti‐CA12 (15180‐1‐AP) antibody was purchased from Proteintech; anti‐*β*‐actin (66009‐1‐IG) antibody was purchased from Proteintech.

### Cell Proliferation Assay, Colony Formation Assay, Wound‐healing Assay and Transwell Assay

Cell viability was measured by using a CellTiter 96 AQueous one solution cell proliferation assay kit (Promega) following the manufacturer's protocol. Briefly, cells were transfected and maintained in stripping medium (phenol red free) for 2 days before treating with estrogen (E_2_, 10^−7^ m) for different time points followed by cell proliferation assay. For lenti‐viral infection, cells were infected with virus for 24 h in normal growth medium then change to stripping medium for 48 h before treating with estrogen (E_2_, 10^−7^ m) for different time points followed by cell proliferation assay. To measure cell viability, 20 µL of CellTiter 96 AQueous one solution reagent was added per 100 µL of culture medium, and the culture plates were incubated for 1–2 h at 37 °C in a humidified, 5% CO_2_ atmosphere incubator. Data was recorded at wavelength 490 nm using a Thermo Multiskan MK3 Microplate Reader.

For colony formation assay, siRNA‐transfected or lenti‐viral‐infected cells were re‐seeded in in 6‐well plates at a density 2000 cells per well and maintained in growth medium. Colonies were examined 10 days after. Briefly, colonies were fixed with methanol/acetic acid solution (3:1) for 5 min and stained with 0.1% crystal violet for 15 min. After washing with PBS extensively, colonies were photographed. Crystal violet was then eluted with 10% acetic acid and measured at wavelength 590 nm using a Thermo Multiskan MK3 Microplate Reader.

For wound‐healing assay, siRNA‐transfected or lenti‐viral‐infected cells were re‐seeded at confluence in 6‐well plates in serum‐free stripping medium and treated with estrogen (E_2_, 10^−7^ m), and wounds were created with a P200 pipette tip. After removing cellular debris by washing cells with PBS, three images of each well were taken. The wounded area was measured by using image J and recorded as A0. The cells were then allowed to migrate back into the wounded area, and images were taken to measure the wounded area 48 h later and recorded as A1. Cell migration was presented as wound closure (%) = (wounded area (A0 − A0 or A1)/wounded area A0) × 100%.

For Transwell assay, siRNA‐transfected or lenti‐viral‐infected cells were re‐seeded on the top compartment of Transwell Boyden chambers (8 µm, Corning, USA) in serum‐free media, and then allowed to migrate to the lower compartment contained complete media with 10% FBS in a humidified, 5% CO_2_ atmosphere incubator at 37 °C. After 36 h, the inserts were washed with PBS and fixed with 4% paraformaldehyde for 15 min at 4 °C and stained with 0.1% crystal violet for 10 min. After washing with PBS extensively, cells that did not migrate into the lower compartment were wiped away with a cotton swab and migrated cells were photographed and counted.

### Xenograft Assay in Nude Mice

Female BALB/C nude mice (age 4–6 weeks) used for xenograft models were housed in the Animal Facility at Xiamen University under pathogen‐free conditions, following the protocol approved by the Animal Ethics Committee of Xiamen University (XMULAC20170038).

For xenograft assay, 5 × 10^6^ MCF7 cells transfected with shCTL or shLINC02568 were suspended in PBS and subcutaneously implanted to nude mice. Nude mice were brushed with or without estrogen (10^−2^ m) every 2 days for the duration of the experiments to sustain tumor growth. All mice were euthanized 8 weeks after subcutaneous injection. Tumors were then excised, photographed, and weighted.

For ASO treatment in vivo, 5 × 10^6^ MCF7 cells were subcutaneously implanted to nude mice, and brushed with estrogen (E_2_, 10^−2^ m) on the neck every 2 days until tumor size reached approximately 100 mm^3^. Mice were then randomly assigned into two groups and ASO were intratumorally injected every 2 days at a dose of 5 nmol per injection (50 µL, 0.1 m in PBS) for eight times, or intravenously injected every 2 days at a dose of 10 nmol per injection (100 µL, 0.1 m in PBS) for eight times. Tumors were then excised, photographed, and weighted.

For combination of ASO and fulvestrant (ICI) or U‐104 in vivo, 5 × 10^6^ MCF7 cells were injected subcutaneously into nude mice, and brushed with estrogen (E_2_, 10^−2^ m) on the neck every 2 days until tumor size reached approximately 100 mm^3^. Mice were then randomly assigned into four groups as control, ASO‐treated, ICI or U‐104‐treated, and ASO and ICI‐ or ASO and U‐104‐treated. ASO were intratumoral injected every 2 days at a dose of 5 nmol per injection (50 µL, 0.1 m in PBS) for six times, ICI were subcutaneous injected once a week at a dose of 250 mg kg^−1^, and U104 were intraperitoneal injected every other day at a dose of 50 mg kg^−1^. Tumors were then excised, photographed, and weighted.

### Isolation of Cytoplasm and Nuclear RNA

Cells in culture (15 cm plate) were washed twice with ice‐cold PBS and then scraped gently into falcon tube (15 mL). Cell pellet were resuspended in 1 mL ice cold buffer containing 10 mm HEPES (pH 8.0), 1.5 µm MgCl_2_, 10 mm KCl, and 1 µm DTT, and then incubated for 15 min on ice to allow cells to swell. Suspension was added with 1% NP‐40 and vortexed 10 s followed by centrifuge 2–3 min at maximum speed. The supernatant was transferred as cytoplasm fraction and the pellet was nuclear fraction. RNA was then extracted using Trizol (Takara) following the manufacturer's protocol.

### Competitive Endogenous RNA Network Analysis

To construct ceRNA network, three different algorithms, TarPmiR,^[^ ^60^ ^]^ miRand,^[^ ^61^ ^]^ and RNAhybrid,^[^ ^62^ ^]^ were utilized to predict miRNAs interacting with LINC02568. The miRNAs that were commonly predicted were chosen for downstream analysis. mRNA targets that these three miRNAs could target to were predicted with Targetscan. To construct the ceRNA network for LINC02568, only the mRNA targets that were shown to be positively regulated by LINC02568 were kept. The ceRNA network was constructed by Cytoscape.^[^ ^63^ ^]^

### Luciferase Reporter Assay

For luciferase reporter assay to determine the interaction between miRNA and target genes, LINC02568 and the 3′UTR of *ESR1* with miR‐1233‐5p binding site as well as the mutant forms were transfected into MCF7 cells for 48 h followed by dual‐luciferase reporter assay. Luciferase activity was determined by Dual Luciferase Assay Kit (Promega) in line with the manufacturer's instructions.

### Chromatin immunoprecipitation Assay

For ChIP (chromatin immunoprecipitation) assay, cells were fixed with 1% formaldehyde (Sigma) for 15 min at room temperature. Fixation was stopped by glycine (0.125 m) followed by washing with PBS twice. Cells were collected and lysed by adding lysis buffer (1% SDS, 10 mm EDTA (pH 8.0) and 50 mm Tris‐Cl (pH 7.8)). Chromatin DNA was sheared to 300–500 bp average in size through sonication. Resultant was immunoprecipitated with control IgG or anti‐RNA polymerase II antibodies overnight at 4 °C, followed by incubation with protein G magnetic beads (BioRad) for 4 h at 4 °C. Beads were then washed twice with TSE1 (0.1% SDS, 1% TritonX‐100, 2 mm EDTA (pH 8.0), 150 mm NaCl, and 20 mm Tris‐Cl (pH 7.8)), TSE2 (0.1% SDS, 1% TritonX‐100, 2 mm EDTA (pH 8.0), 400 mm NaCl, and 20 mm Tris‐Cl (pH 7.8)) followed by washing with TE buffer (50 mm Tris‐Cl (pH 8.0) and 10 mm EDTA (pH 8.0)) twice. The protein–DNA complex was de‐cross‐linking by heating at 65 °C overnight with TE/SDS buffer (TE buffer with 1% SDS). Immunoprecipitated DNA was purified by using QIAquick spin columns (Qiagen) and subjected to qPCR analysis. The primer used to detect the binding of RNA polymerase II on CA12 promoter region were presented in Table S6 (Supporting Information).

### Intracellular and Extracellular pH Measurement

For intracellular pH measurement, siRNA‐transfected or lenti‐viral‐infected cells were re‐seeded in 96 well plate. Cells were washed twice with HEPES buffer (150 mm NaCl, 5 mm KCl, 5 mm glucose, and 20 mm HEPES (pH 7.4)). Cells were incubated in HEPES buffer containing pH sensitive dye, 2′,7′‐Bis‐(2‐carboxyethyl)−5‐(and‐6)‐carboxyfluorescein acetoxymethyl ester (BCECF‐AM, Beyotime), at a final concentration of 5 µm at 37 °C for 30 min in dark. Cells were then washed twice with HEPES buffer and the fluorescence intensities were examined using Multimode Reader (Tecan spark) at the excitation wavelength of 488 nm and emission wavelength of 530 nm and 640 nm. The ratio of emission intensities at 530 nm versus that of 640 nm were transformed into intracellular pH based on the standard curve as determined by in vivo calibration. The in vivo calibration was performed with the ionophore nigericin (Sigma) at a final concentration of 10 µg mL^−1^ diluted in a set of high K^+^ phosphate buffer (25 mm HEPES, 145 mm KCl, 0.8 mm MgCl_2_, 1.8 mm CaCl_2_, and 5 mm glucose) of determined pH ranging of 6.6, 7.0, 7.2, 7.4, 7.8, and 8.2.

For extracellular pH measurement, the culture medium was collected and measured by the pH microelectrode meter (pH500, CLEAN).

### Extracellular Acidification Rate Measurement

ECAR was measured in MCF7 cells by using a Seahorse XF Cell Glycolysis Stress Test Kit (Agilent) in an Extracellular Flux Analyzer XFe96 (Seahorse Bioscience). Specifically, siRNA‐transfected or lenti‐viral‐infected MCF7 cells were re‐seeded at approximately 10000 cells per well onto a Seahorse 96‐well assay plate and incubated in XF DMEM Medium without bicarbonate, glucose, pyruvate, or glutamine (pH 7.4) (Seahorse Bioscience) in an incubator for 1 h (37 °C without CO_2_). Then, the pre‐warmed glucose, oligomycin, and 2‐DG were sequentially added into the injector (glucose, 10 mm; oligomycin, 1 µm; 2‐DG, 50 mm). ECAR was measured three times for 6 min each, with 3 min of measuring time and 3 min of mixing time between measurements. The data obtained were used to evaluate the glycolytic function include glycolysis, glycolytic capacity, glycolytic reserve, and nonglycolytic acidification.

### Statistical Analysis

Student's *t*‐tests were used for comparisons between experimental and control conditions, and two‐way analysis of variance (ANOVA) was used for multiple group comparisons. Results from xenograft experiments and clinical breast samples were analyzed by GraphPad Prism 7. The results were expressed as the mean ± standard error of mean (SEM) of at least three independent experiments. All statistical analyses were performed using two‐tailed *p* values.

## Conflict of Interest

The authors declare no conflict of interest.

## Author Contributions

X.C., J.‐C.D. contributed equally to this work. W.L. and X.C. conceived the original ideas, designed the project, and wrote the manuscript with inputs from H.H. and G.T. X.C. performed the majority of the experiments with participation from X.S., Y.L., J.D., Z.W., and J.L., and J.D., and G.H. performed the bioinformatics analyses. All of the authors discussed and commented on the study. All authors read and approved the final manuscript.

## Supporting information

Supporting Information

Supplemental Table 1

Supplemental Table 2

Supplemental Table 3

Supplemental Table 4

Supplemental Table 5

Supplemental Table 6

Supplemental Table 7

## Data Availability

The data that support the findings of this study are available in the supplementary material of this article.
