# Supplementary material for: Estrogen‐Induced LncRNA, LINC02568, Promotes Estrogen Receptor‐Positive Breast Cancer Development and Drug Resistance Through Both In Trans and In Cis Mechanisms
Source: Adv Sci (Weinh). 2023 Jul 5;10(25):2206663. doi: 10.1002/advs.202206663 (PMC10477896; doi:10.1002/advs.202206663)
Supplement: Supplementary file 1 — Supporting Information [file ADVS-10-2206663-s004.pdf]

ADVANCED SCIENCE
Open Access

Supporting Information

for *Adv. Sci.*, DOI 10.1002/advs.202206663

Estrogen-Induced LncRNA, LINC02568, Promotes Estrogen Receptor-Positive Breast Cancer Development and Drug Resistance Through Both *In Trans* and *In Cis* Mechanisms

*Xue Chen, Jian-cheng Ding, Guo-sheng Hu, Xing-yi Shu, Yan Liu, Jun Du, Zi-jing Wen, Jun-yi Liu, Hai-hua Huang, Guo-hui Tang and Wen Liu**

Supplementary Information

Supplementary figure legends

Figure S1. Estrogen induces a large number of lncRNAs in ER^+ breast cancer cells

(A) The correlation of the effects of estrogen (E_2) on whole transcriptome based on RNA-seq between two biological repeats is shown.

(B) The number of lncRNAs regulated by E_2, both up- and down-regulated, in at least three out of five ER^+ breast cancer cell lines based on RNA-seq analysis, including CAMA-1, EFM-19, HCC1500, MDA-MB-134-VI, and T-47D ($q < 0.05$).

(C) Breast tumors and the corresponding normal tissues were collected from ER^+ breast cancer patients ($n = 10$) and subjected to RNA extraction and RT-qPCR analysis to examine the expression of lncRNA/mRNA pairs including DSCAM-AS1/DSCAM, PVT1/MYC, IRAIN/IGF1R, LOC100129931/GRPEL1, KCTD21-AS1/GAB2, SLC25A25-AS1/SLC25A25, SH3BP5-AS1/SH3BP5, C15orf59-AS1/C15orf59, LOC90784/POLR1A, and LOC101928837/TSKU as indicated (± s.e.m.).

Figure S2. Estrogen-induced lncRNA, LINC02568, promotes the malignant behaviors of ER^+ breast cancer cell

(A) The expression of LINC02568 in different cancer types in GEPIA2 database is shown. LINC02568 is significantly upregulated in BRCA and downregulated in PAAD and SKCM ($FC \geq 2$, $P \leq 0.01$).

(B-D) Quantification of the crystal violet dye (B), the wound closure (C), and the number of invasive cells (D) as shown in Fig. 2G, Fig. 2H, and Fig. 2I, respectively (± s.e.m., $*P < 0.05$, $**P < 0.01$, $***P < 0.001$).

(E-G) Quantification of the crystal violet dye (E), the wound closure (F), and the number of invasive cells (G) as shown in Fig. 2L, Fig. 2M, and Fig. 2N, respectively (± s.e.m., **P < 0.01, ***P < 0.001).

(H) MCF7 cells were transfected with control siRNA (siCTL) or siRNA specifically targeting LINC02568 (siLINC02568) and maintained in stripping medium for 48 hours before treated with estrogen (E_2, 10^{-7} M, 6h), followed by RNA extraction and RT-qPCR analysis to examine the expression of LINC02568 (± s.e.m., ***P < 0.001).

(I, J, L, M) MCF7 cells were transfected with control siRNA (siCTL) or siRNA specifically targeting LINC02568 (siLINC02568) and maintained in stripping medium for 48 hours before treating with estrogen (E_2, 10^{-7} M) for duration as indicated, followed by cell proliferation assay (I), colony formation assay (J), wound-healing assay (L), and transwell assay (M) (± s.e.m., ***P < 0.001).

(K, N, O) Quantification of the crystal violet dye (K), the wound closure (N), and the number of invasive cells (O) in (J), (L), and (M), respectively, is shown (± s.e.m., ***P < 0.001).

(P, Q, S, U) T47D cells were infected with lenti-virus expressing shCTL or shLINC02568, and maintained in stripping medium for 48 hours before treating with estrogen (E_2, 10^{-7} M) for duration as indicated, followed by cell proliferation assay (P), colony formation assay (Q), wound-healing assay (S), and transwell assay (U) (± s.e.m., *P < 0.05, **P < 0.01).

(R, T, V) Quantification of the crystal violet dye (R), the wound closure (T), and the number of invasive cells (V) in (Q), (S), and (U), respectively, is shown (± s.e.m., ***P

< 0.001).

Figure S3. LINC02568 serves as a miRNA sponge for miR-1233-5p to promote *ESR1* expression

(A) CeRNA network constituting of LINC02568-miRNAs-mRNAs (genes positively-regulated by LINC02568, n = 206) is shown. Nodes in green, yellow, and light blue represent LINC02568, miRNAs, and mRNAs, respectively.

(B) UCSC genome browser view of *ESR1* expression from RNA-seq as described in Fig. 3B is shown.

(C) MCF7 cells were infected with lenti-virus expressing control shRNA (shCTL) or two individual shRNAs specifically targeting LINC02568 (shLINC02568-1 and shLINC02568-2) for 48 hours before treated with estrogen (E_2, 10^{-7} M, 6 h), followed by RNA extraction and RT-qPCR analysis to examine the expression of ERα-target genes (± s.e.m.).

(D) T47D cells were transfected with siCTL or siLINC02568 and maintained in stripping medium for 48 hours before treated with estrogen (E_2, 10^{-7} M, 6 h), followed by RNA extraction and RT-qPCR analysis to examine the expression of ERα-target genes (± s.e.m.).

(E) Breast tumors and the corresponding normal tissues as described in Fig. S1C (n = 10) were subjected to RNA extraction and RT-qPCR analysis to examine the expression of miR-1233-5p (± s.d., ns: non-significant).

(F) Tumor samples as described in Fig. 2O were subjected to RNA extraction and RT-qPCR analysis to examine the expression of miR-1233-5p (± s.d., ns: non-significant).

(G, H) Quantification of the crystal violet dye in Fig. 4J (G) and Fig. 4L (H) is shown (± s.e.m., ***P < 0.001).

Figure S4. CA12 is highly expressed in ER$^+$ breast tumor samples, and its expression is highly correlated with that of LINC02568

(A, B) Tumor samples as described in Fig. 2O were subjected to RNA extraction and RT-qPCR analysis to examine the expression of *CA12* (A) and pre-mRNA of *CA12* (B) (± s.d., *P < 0.05).

(C) The expression of CA12 in different subtypes of breast tumors in TCGA portal database is shown (http://tumorsurvival.org/).

(D) Breast tumors and the corresponding adjacent and normal tissues as described in Fig.2C were subjected to RNA extraction and RT-qPCR analysis to examine the expression of CA12 (± s.d., ***P < 0.001, ns: non-significant).

(E) Different breast cancer cell lines as indicated were subjected to RNA extraction and RT-qPCR analysis to examine the expression of CA12 (± s.e.m.).

(F) The correlation between the expression of LINC02568 and CA12 in ER$^+$ breast tumor samples in TANRIC is shown.

(G) The correlation between the expression of LINC02568 and CA12 in ER$^+$ breast tumors as described in Fig. 2C.

(H) MCF7 cell were cultured in stripping medium for 48 hours and treated with estrogen (E_2, 10^{-7} M) for 6 hours, followed by RNA extraction and RT-qPCR analysis to examine the expression of CAs as indicated (± s.e.m.).

Figure S5. LINC02568 regulates CA12 to control tumor-specific pH homeostasis

and promote the malignant behaviors of ER^+ breast cancer cell

(A, B, C) Quantification of the crystal violet dye (A), the wound closure (B), and the number of invasive cells (C) in Fig. 5J, Fig. 5K, and Fig. 5L, respectively, is shown (± s.e.m., $**P < 0.01$, $***P < 0.001$).

(D-G) MCF7 cells were transfected with siCTL or siRNA specifically targeting CA12 (siCA12) for 48 hours, followed by RNA extraction and RT-qPCR analysis (D), immunoblotting analysis (E), intracellular pH measurement (F) and extracellular pH measurement (G) (± s.e.m., $**P < 0.01$, $***P < 0.001$).

(H, I, K, M) MCF7 cells were transfected with siCTL or siRNA specifically targeting CA12 (siCA12), followed by cell proliferation assay (H), colony formation assay (I), wound-healing assay (K), and transwell assay (M) (± s.e.m., $**P < 0.01$, $***P < 0.001$).

(J, L, N) Quantification of the crystal violet dye (J), the wound closure (L), and the number of invasive cells (N) in (I), (K), and (M), respectively, is shown (± s.e.m., $**P < 0.01$, $***P < 0.001$).

(O, P) MCF7 cells were infected with lenti-virus expressing control vector or CA12 for 48 hours followed by immunoblotting analysis (O) and extracellular pH measurement (P) (± s.e.m., $***P < 0.001$).

(Q, R, T, V) MCF7 cells were infected with lenti-virus expressing control vector or CA12 followed by cell proliferation assay (Q), colony formation assay (R), wound-healing assay (T), and transwell assay (V) (± s.e.m., $*P < 0.05$, $**P < 0.01$).

(S, U, W) Quantification of the crystal violet dye (S), the wound closure (U), and the number of invasive cells (W) in (R), (T), and (V), respectively, is shown (± s.e.m., **P

< 0.01, ***P < 0.001).

(X, Y) MCF7 cells were transfected with siCTL and siLINC02568 for 48 hours, followed by intracellular (X) and extracellular (Y) pH measurement (± s.e.m., **P < 0.01, ***P < 0.001).

(Z) MCF7 cells were infected with lenti-virus expressing control vector or LINC02568 for 48 hours, followed by extracellular pH measurement (± s.e.m., ***P < 0.01).

(X', Y') MCF7 cells transfected with siCTL or siLINC02568 (X') or infected with lenti-virus expressing control vector or LINC02568 (Y') for 24 hours were re-seeded onto a Seahorse 96-well assay plate (approximately 10,000 cells per well). Cells were subjected to extracellular acidification rate measurement 24 hours later (± s.e.m.).

(Z', X'', Y'') Quantification of the crystal violet dye in Fig. 5S (Z'), the wound closure in Fig. 5T (X''), and the number of invasive cells in Fig. 5U (Y''), respectively, is shown (± s.e.m., **P<0.01, ***P<0.001).

Figure S6. ASO targeting LINC02568 is potent in suppressing ER^+ breast tumor growth

(A, B, C) Quantification of the crystal violet dye (A), the wound closure (B), and the number of invasive cells (C) in Fig. 6C, Fig. 6D, Fig. 6E, respectively, is shown (± s.e.m., ***P < 0.001).

(D) Tumors as described in Fig. 6F were subjected to examine the expression of miR-1233-5p (± s.d., ns: non-significant).

(E) MCF7 cells were injected subcutaneously into female BALB/C nude mice, and brushed with estrogen (E_2, 10^{-2} M) on the neck every 2 days until tumor size reached

approximately 100 mm^3. Mice were then randomly assigned into two groups (n = 3), and injected intravenously with ASO three times a week at a dose of 10 nmol/injection (100 μl, 100 mM in PBS) for 8 times. Tumors were harvested, photographed, and weighted.

(F) The weight of tumors in (E) is shown (± s.d.).

(G) Quantification of the crystal violet in Fig. 6L is shown (± s.e.m., ***P < 0.001).

(H) MCF7 cells were transfected with ASO-CTL or ASO-LINC02568 and maintained in stripping medium for 48 hours before treated with estrogen (E_2, 10^{-7} M, 6 h) in the presence or absence of tamoxifen (5 μM, 6 h), followed by RNA extraction and RT-qPCR analysis to examine the expression of LINC02568, *CA12*, and representative ERα-target genes as indicated (± s.e.m.).

(I, J) MCF7 cells were transfected with ASO-CTL or ASO-LINC02568 and treated with or without tamoxifen (5 μM, 6 h) for duration as indicated, followed by cell proliferation assay (I) and colony formation assay (J) (± s.e.m., **P < 0.01, ***P < 0.001).

(K) Quantification of the crystal violet dye in (J) is shown (± s.e.m., ***P < 0.001).

(L) Quantification of the crystal violet dye in Fig. 6R is shown (± s.e.m., ***P < 0.001).

(M, N) MCF7 cells were treated with or without tamoxifen (5 μM) or U-104 (50 μM) for duration as indicated followed by cell proliferation assay (M) and colony formation assay (N) (± s.e.m., **P < 0.01, ***P < 0.001).

(O) Quantification of the crystal violet dye in (N) is shown (± s.e.m., ***P < 0.001).

Supplementary table legends

Table S1. Estrogen-induced lncRNAs identified through transcriptome analysis in ER^+ breast cancer cells.

Table S2. Estrogen-induced lncRNAs identified in MCF7 cells ($q < 0.05$, $FC > 1.5$, $n = 129$) as shown in Fig. 1B was interrogated into GEPIA2 database. The fold change and significance of lncRNA expression in ER^+ breast tumor tissue compared to normal tissues is shown.

Table S3. The expression of LINC02568-positively regulated genes that are included in the ceRNA network in ER^+ breast tumor tissues.

Table S4. The correlation between the expression of LINC02568 and LINC02568-positively regulated genes included in the ceRNA network in ER^+ breast tumor tissues.

Table S5. Genes that are positively-correlated with LINC02568 in ER^+ breast tumor samples.

Table S6. Sequence information for all primers used in the current study. Sequence information of qPCR primers designed to detect the expression of mRNA or lncRNA as indicated and factor binding on promoter region (ChIP), and primers used for molecular cloning are shown. F: forward; R: reverse.

Table S7. Sequence information for shRNAs, siRNAs, and ASOs used in the current study.

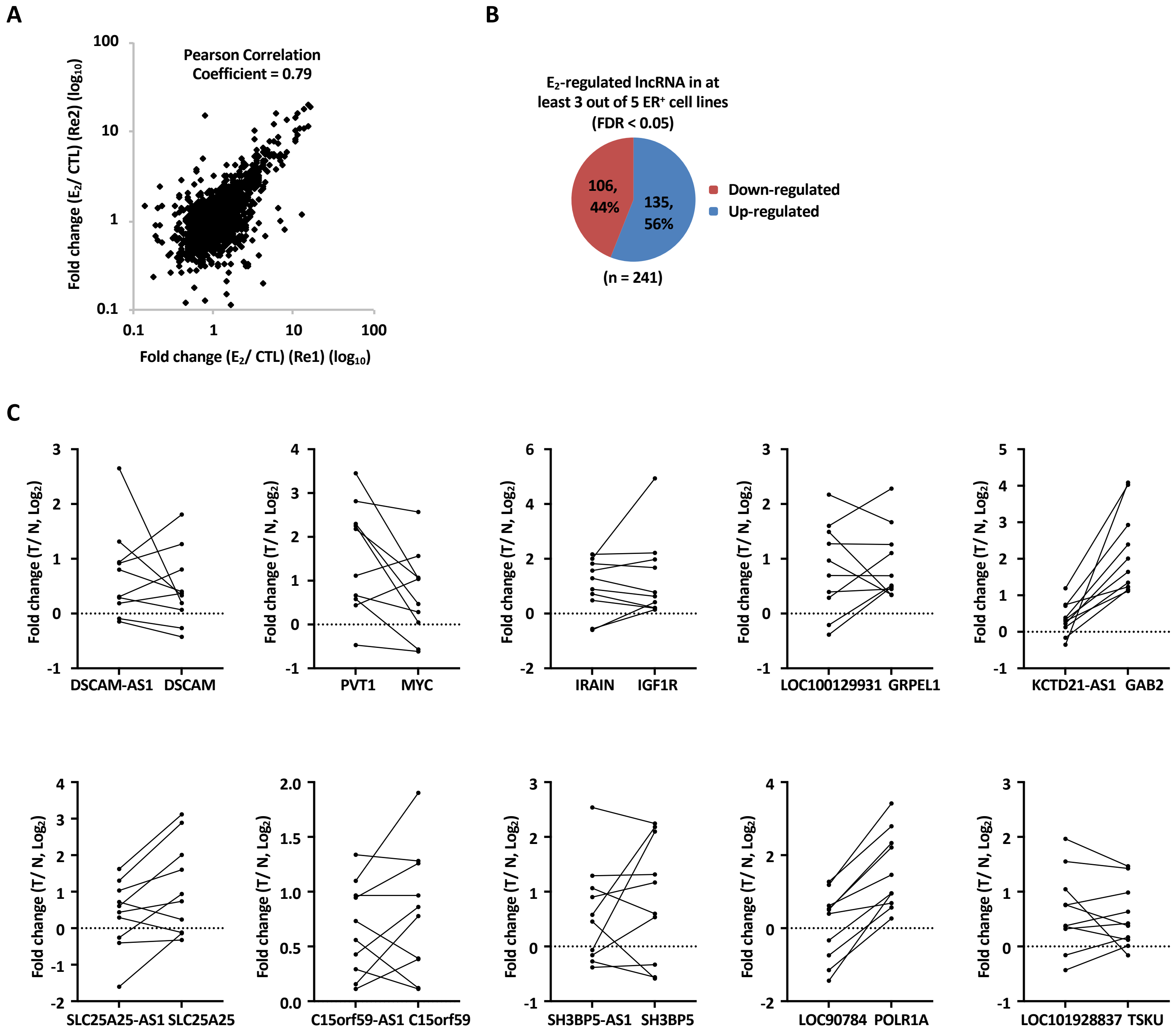
A
B
C
Pearson Correlation Coefficient = 0.79
Fold change (E2/ CTL) (Re2) (log10)
Fold change (E2/ CTL) (Re1) (log10)
E2-regulated lncRNA in at least 3 out of 5 ER+ cell lines (FDR < 0.05)
106, 44%
135, 56%
Down-regulated
Up-regulated
(n = 241)
Fold change (T/ N, Log2)
DSCAM-AS1 DSCAM
PVT1 MYC
IRAIN IGF1R
LOC100129931 GRPEL1
KCTD21-AS1 GAB2
SLC25A25-AS1 SLC25A25
C15orf59-AS1 C15orf59
SH3BP5-AS1 SH3BP5
LOC90784 POLR1A
LOC101928837 TSKU

Figure S1

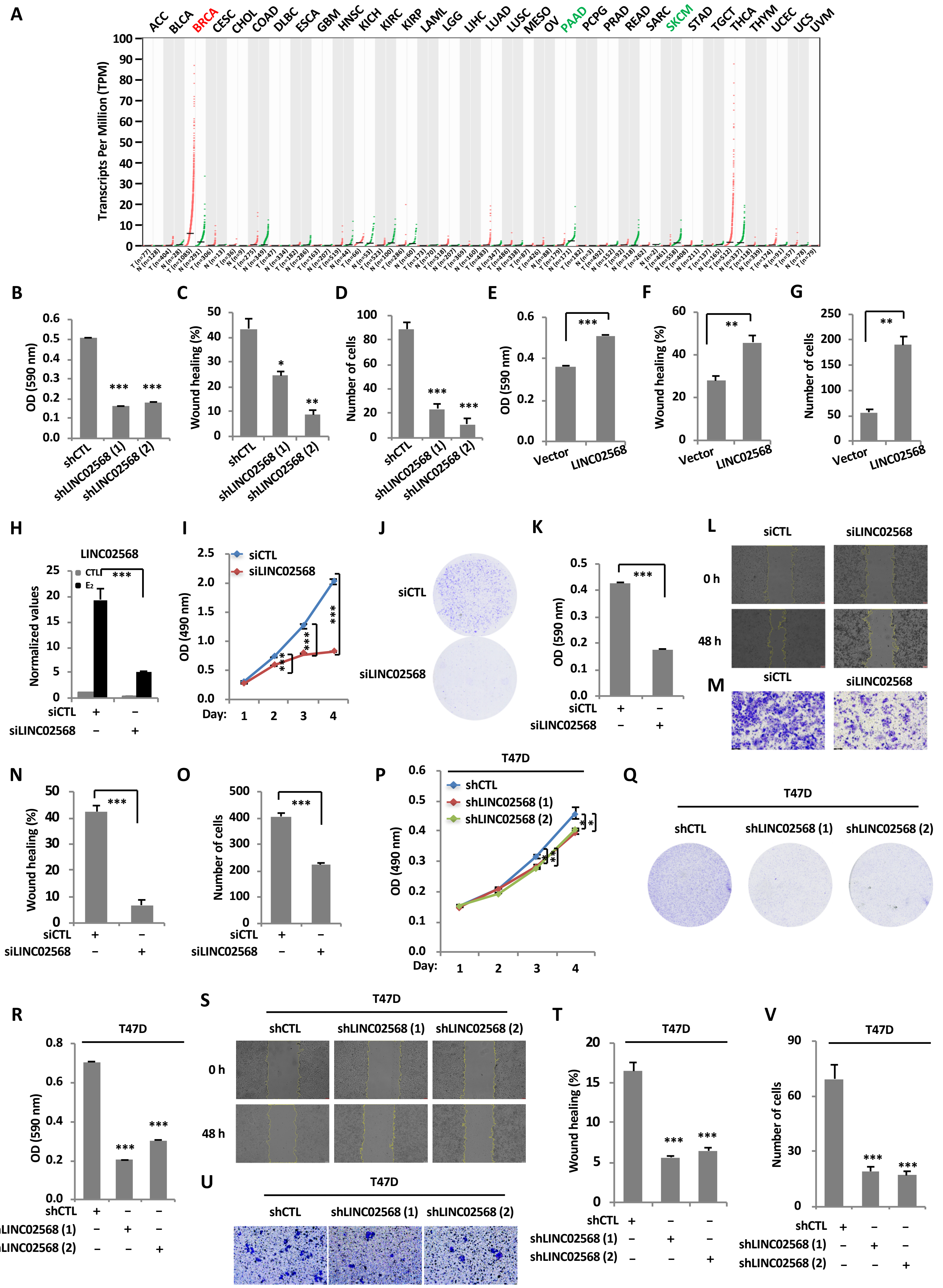
A
ACC BLCA BRCA CESC CHOL COAD DLBC ESCA GBM HNSC KICH KIRC KIRP LAML LGG LIHC LUAD LUSC MESO OV PAAD PCPG PRAD READ SARC SKCM STAD TGCT THCA THYM UCEC UCS UVM
Transcripts Per Million (TPM)
B
OD (590 nm)
shCTL
shLINC02568 (1)
shLINC02568 (2)
C
Wound healing (%)
D
Number of cells
E
Vector
LINC02568
F
G
H
LINC02568
Normalized values
CTL
E2
siCTL
siLINC02568
I
OD (490 nm)
Day:
J
K
L
0 h
48 h
M
N
O
P
T47D
Q
R
S
T
U
V

Figure S2

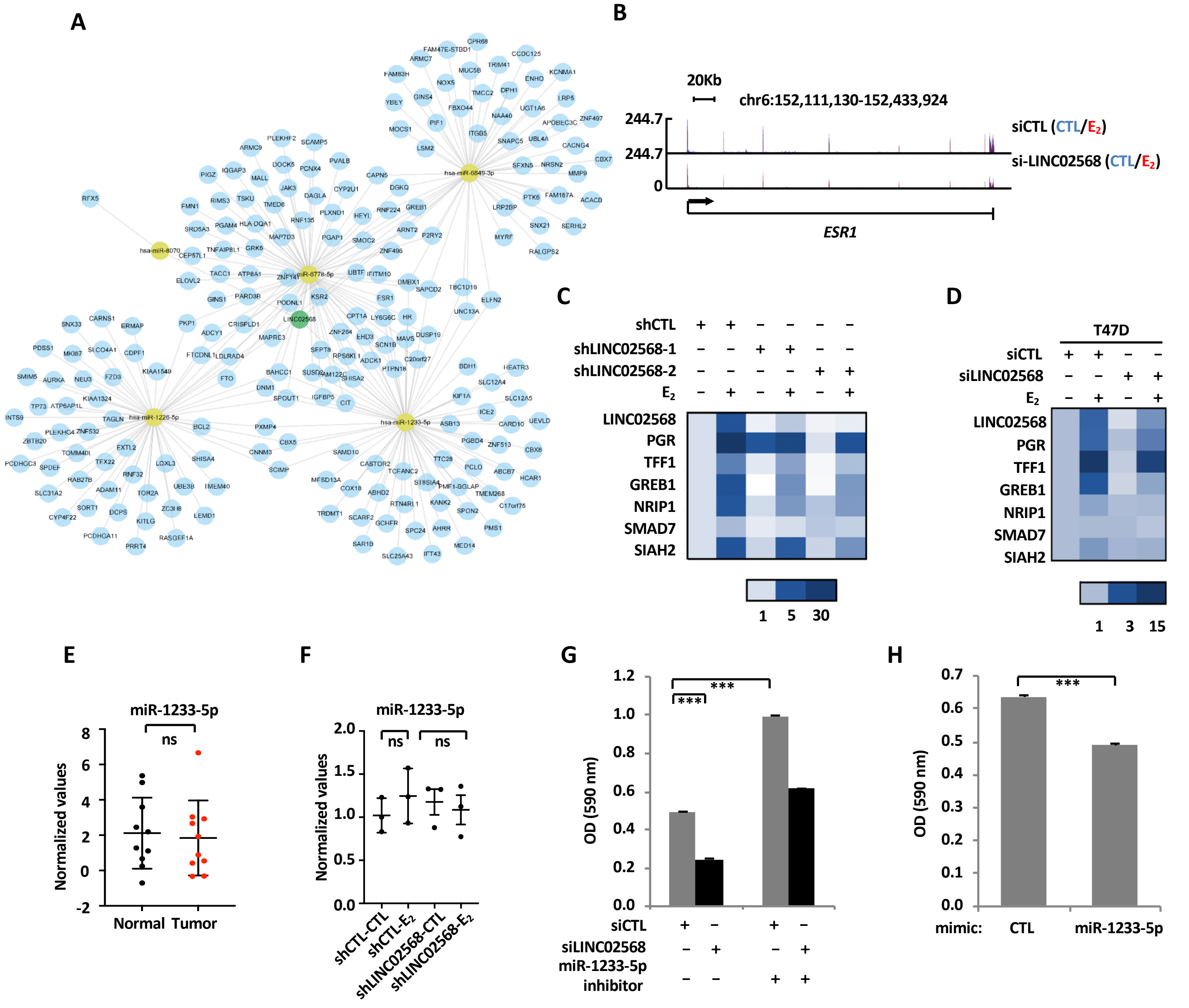
A
B
20Kb
chr6:152,111,130-152,433,924
244.7
siCTL (CTL/E2)
si-LINC02568 (CTL/E2)
0
ESR1
C
shCTL
shLINC02568-1
shLINC02568-2
E2
LINC02568
PGR
TFF1
GREB1
NRIP1
SMAD7
SIAH2
1 5 30
D
T47D
siCTL
siLINC02568
1 3 15
E
miR-1233-5p
Normalized values
Normal Tumor
F
shCTL-CTL
shCTL-E2
shLINC02568-CTL
shLINC02568-E2
G
OD (590 nm)
siCTL
siLINC02568
miR-1233-5p inhibitor
H
mimic:
CTL
miR-1233-5p

Figure S3

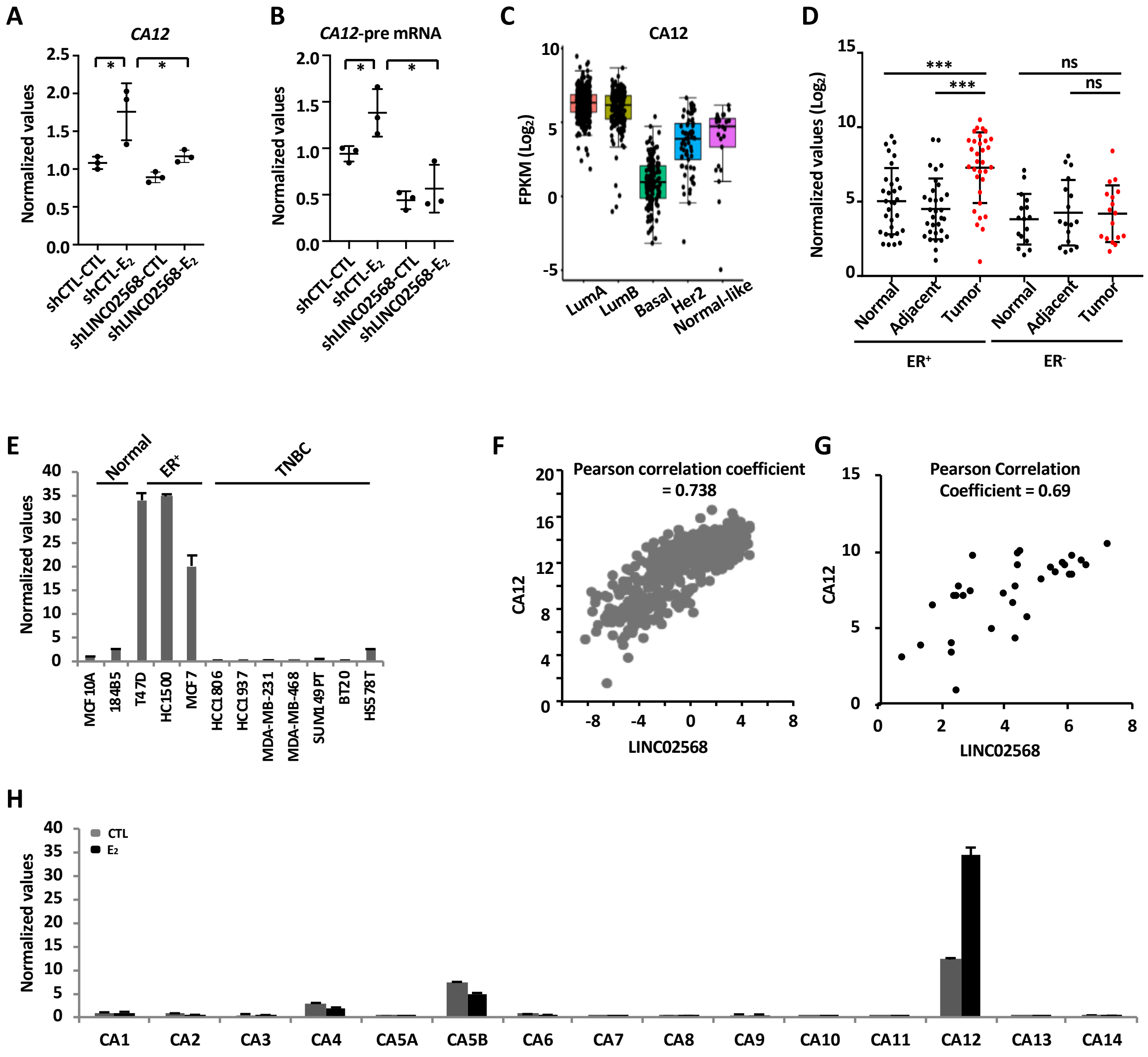

A
CA12
Normalized values
shCTL-CTL
shCTL-E2
shLINC02568-CTL
shLINC02568-E2
B
CA12-pre mRNA
C
CA12
FPKM (Log2)
LumA
LumB
Basal
Her2
Normal-like
D
Normalized values (Log2)
***
ns
Normal
Adjacent
Tumor
ER+
ER-
E
Normal
ER+
TNBC
MCF10A
184B5
T47D
HC1500
MCF7
HCC1806
HCC1937
MDA-MB-231
MDA-MB-468
SUM149PT
BT20
HS578T
F
Pearson correlation coefficient
= 0.738
CA12
LINC02568
G
Pearson Correlation
Coefficient = 0.69
H
CTL
E2
CA1
CA2
CA3
CA4
CA5A
CA5B
CA6
CA7
CA8
CA9
CA10
CA11
CA12
CA13
CA14

Figure S4

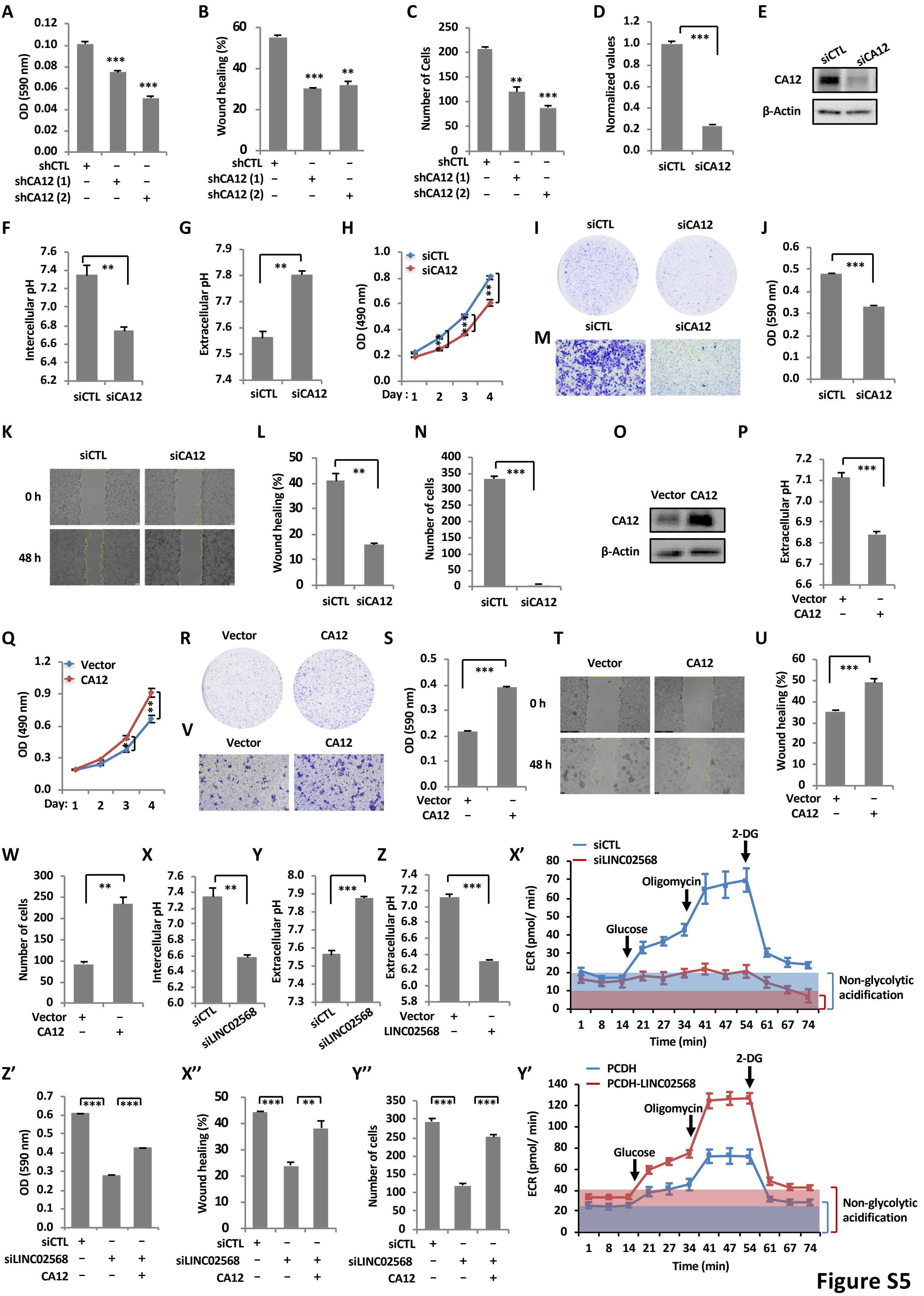
A
B
C
D
E
F
G
H
I
J
K
L
M
N
O
P
Q
R
S
T
U
V
W
X
Y
Z
X'
Z'
X''
Y''
Y'
OD (590 nm)
Wound healing (%)
Number of Cells
Normalized values
Intercellular pH
Extracellular pH
OD (490 nm)
Number of cells
ECR (pmol/ min)
shCTL
shCA12 (1)
shCA12 (2)
siCTL
siCA12
CA12
β-Actin
Day :
0 h
48 h
Vector
siLINC02568
LINC02568
Glucose
Oligomycin
2-DG
Non-glycolytic acidification
Time (min)
PCDH
PCDH-LINC02568

Figure S5

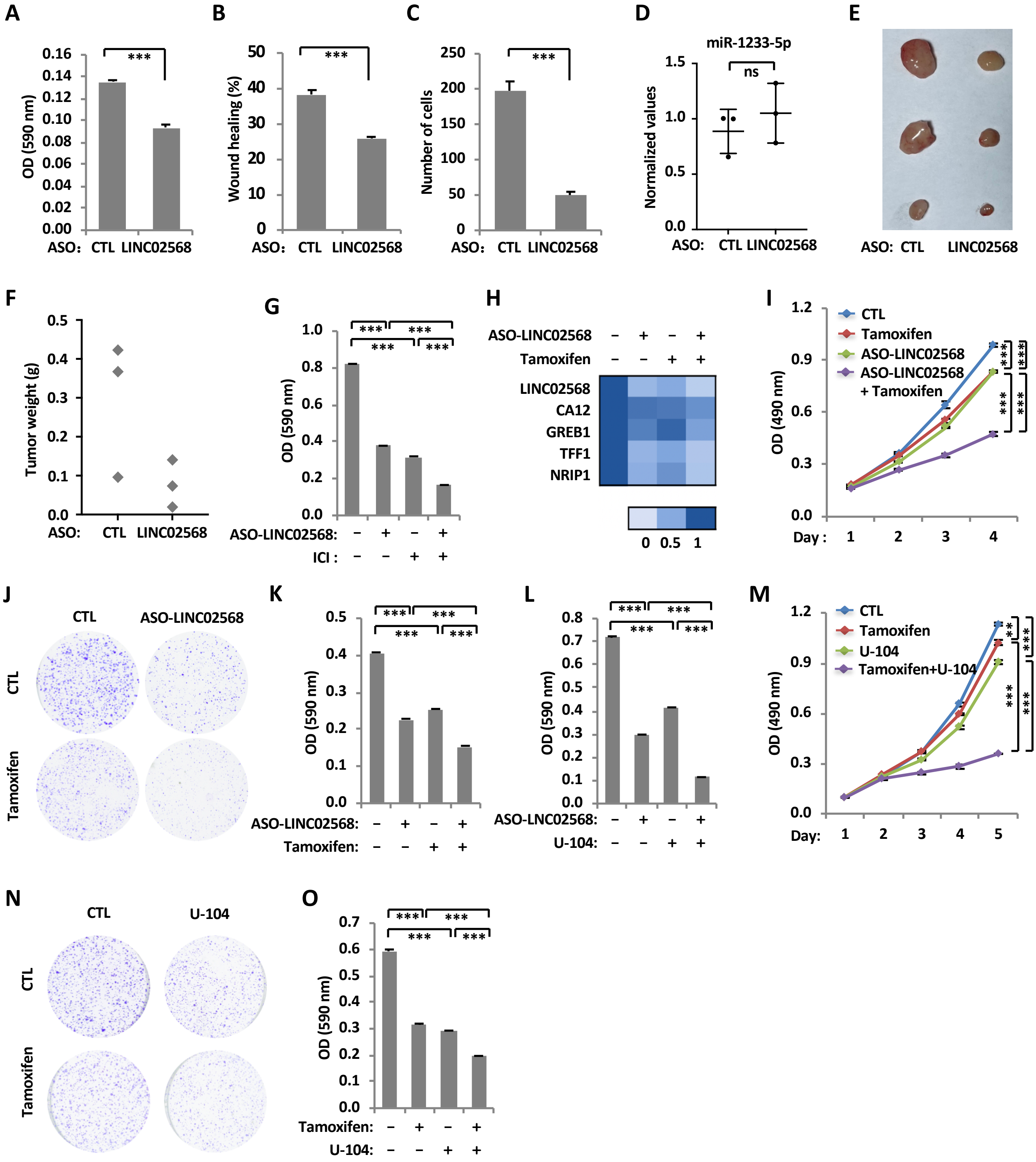
A
OD (590 nm)
ASO: CTL LINC02568
B
Wound healing (%)
ASO: CTL LINC02568
C
Number of cells
ASO: CTL LINC02568
D
miR-1233-5p
ns
Normalized values
ASO: CTL LINC02568
E
ASO: CTL LINC02568
F
Tumor weight (g)
ASO: CTL LINC02568
G
OD (590 nm)
ASO-LINC02568:
ICI :
H
ASO-LINC02568
Tamoxifen
LINC02568
CA12
GREB1
TFF1
NRIP1
0 0.5 1
I
CTL
Tamoxifen
ASO-LINC02568
ASO-LINC02568 + Tamoxifen
OD (490 nm)
Day :
J
CTL
ASO-LINC02568
Tamoxifen
K
OD (590 nm)
ASO-LINC02568:
Tamoxifen:
L
OD (590 nm)
ASO-LNC02568:
U-104:
M
CTL
Tamoxifen
U-104
Tamoxifen+U-104
OD (490 nm)
Day:
N
CTL
U-104
Tamoxifen
O
OD (590 nm)
Tamoxifen:
U-104:

Figure S6
